# Laser Surface Alloying of Austenitic 316L Steel with Boron and Some Metallic Elements: Microstructure

**DOI:** 10.3390/ma13214852

**Published:** 2020-10-29

**Authors:** Michał Kulka, Daria Mikołajczak, Natalia Makuch, Piotr Dziarski, Damian Przestacki, Dominika Panfil-Pryka, Adam Piasecki, Andrzej Miklaszewski

**Affiliations:** 1 Institute of Materials Science and Engineering, Poznan University of Technology, Pl. M.Sklodowskiej-Curie 5, 60-965 Poznan, Poland; natalia.makuch@put.poznan.pl (N.M.); piotr.dziarski@put.poznan.pl (P.D.); dominika.panfil-pryka@put.poznan.pl (D.P.-P.); adam.piasecki@put.poznan.pl (A.P.); andrzej.miklaszewski@put.poznan.pl (A.M.); 2 WSK Poznan Ltd., Unii Lubelskiej Street 3, 61-249 Poznan, Poland; daria.mikolajczak02@gmail.com; 3 Institute of Mechanical Technology, Poznan University of Technology, Piotrowo Street 3, 60-965 Poznan, Poland; damian.przestacki@put.poznan.pl

**Keywords:** laser surface alloying, laser boriding, 316L steel, microstructure, composite surface layer, phase analysis, chemical composition, thickness of laser-alloyed layer, dilution ratio

## Abstract

Austenitic 316L steel is known for its good oxidation resistance and corrosion behavior. However, the poor wear protection is its substantial disadvantage. In this study, laser surface alloying with boron and some metallic elements was used in order to form the surface layers of improved wear behavior. The microstructure was studied using OM, SEM, XRD, and EDS techniques. The laser-alloyed layers consisted of the only re-melted zone (MZ). The hard ceramic phases (Fe_2_B, Cr_2_B, Ni_2_B, or Ni_3_B borides) occurred in a soft austenitic matrix. The relatively high overlapping (86%) resulted in a uniform thickness and homogeneous microstructure of the layers. All the laser-alloyed layers were free from defects, such as microcracks or gas pores, due to the use of relatively high dilution ratios (above 0.37). The heat-affected zone (HAZ) wasn’t visible in the microstructure because of the extended stability of austenite up to room temperature and no possibility to change this structure during fast cooling. The use of the mixtures of boron and selected metallic elements as the alloying materials caused the diminished laser beam power in order to obtain the layers of acceptable quality. The thickness of laser-alloyed layers (308–432 μm) was significantly higher than that produced using diffusion boriding techniques.

## 1. Introduction

AISI 316L austenitic stainless steel is well-known for its excellent resistance to oxidation and good corrosion behavior. Hence, this steel is often used in the aggressively corrosive environment in nuclear reactor applications as well as at high-temperature conditions. However, the important disadvantage of this steel is a relatively low hardness (about 200 HV). It results in poor wear resistance and is the reason for the limited use of 316L steel. There is no possibility to harden the austenitic structure by the conventional heat treatment because of the extended stability of this phase up to room temperature [1]. Therefore, the formation of surface layers has essential importance if the austenitic steel has to be more protected against wear without sacrificing corrosion resistance. The typical thermochemical treatment, usually improving the wear behavior of the constructional or tool steels, such as nitriding, carburizing, or boriding, could be used for the austenitic steel under certain conditions. These processes weren’t effective because of the easy passivation of austenitic steel. The layer of oxides hampered the absorption and the diffusion of interstitial elements, such as nitrogen, carbon, or boron. Therefore, appropriate surface activation was necessary before the typical thermochemical treatment. First of all, the oxides were removed mechanically or chemically from the surface. Hence, the physical techniques under glow discharge conditions were intensively developed for the formation of the surface layers, which could improve the tribological properties of austenitic steel [2,3,4,5,6,7,8,9,10,11,12,13,14,15,16,17,18,19,20,21,22,23,24,25,26,27,28,29,30,31]. These techniques were often called also plasma or ion processes [32]. Their main advantages were as follows: the reduction of the process temperature and the possibility of sputter cleaning of the surface during the first stage of the process. It allowed us to remove the oxides from the surface.

AISI 316L steel was often subjected to glow discharge-assisted low-temperature gas nitriding [2,3,4,5,6,7,8,9,10,11,12,13]. Such a process, carried out at 440 °C (713 K) [2] or at 450 °C (723 K) [3] for 6 h, resulted in producing the relatively thin layer (4 μm) consisting of chromium nitrides (CrN) as well as of S-phase, also called expanded austenite (γ_N_), i.e., austenite supersaturated with nitrogen. Low-temperature plasma gas nitriding (LTPGN) could also provide the nitrided layers, which were composed of expanded austenite (γ_N_) as well as iron nitrides γ’—Fe_4_N and ε—Fe_2-3_N [4], Fe_4_N, Fe_3_N, and Fe_2_N [5], or only Fe_4_N [6] without CrN phase. The thickness of the layer ranged from 6.72 [5] to 14 μm [6]. The higher nitrogen percentage in the atmosphere resulted in the presence of CrN, Fe_3_N, and Fe_4_N nitrides, as well as expanded austenite (γ_N_) in the layer with a thickness of 15 μm [6]. Sometimes, the LTPGN process resulted in the formation of the S-phase only of the thickness equal to 9 μm [7] or in the range of 2–8 [8] and 13–16 μm [9], depending on the process parameters (temperature and time). Beneath the S-phase, the diffusion zone with a thickness of 100 μm was also identified [9]. The use of a so-called active screen during LTPGN advantageously influenced the thickness of the nitrided layer [10,11,12,13]. The short-term (2–6 h) LTPGN processes with the use of an active screen increased the thickness of the nitrided layer from 2 to 6 μm [10] or from 2.51 to 6.34 μm [11]. The sequence of the phases in the nitrided layer from the surface towards the core of 316L steel was as follows: CrN, Cr_2_N, and expanded austenite (γ_N_) [10,11]. The use of an active screen during the long-term LTPGN (for 15–50 h) also influenced the thickness of the layer advantageously [12,13], increasing it from 6 to 15 μm [12] or up to 16.2 μm [13] as a consequence of the process duration of 35 or 30 h, respectively. The nitrided layer was composed of CrN nitrides with S-phase if the process was carried out without active screen, and S-phase only in the case of the use of active screen [12,13]. In general, the use of an active screen led to an increase in the plasma temperature, reducing the density of the substrate and increasing the average free path of electrons and, as a consequence, increasing the energy of ions bombarding the surface [11]. The nitrided layer thickness also increased in 316L steel after high-temperature plasma gas nitriding (HTPGN), which was carried out using the temperature above 480 °C (753 K) [3,5,14,15]. The layer thickness increased to about 20 [3] or 54.7 μm [5] if such a process was carried out for 6 h at 550 °C (823 K) or 520 °C (793 K), respectively. CrN and Fe_4_N nitrides, as well as S-phase [3] or only the chromium and iron nitrides (Cr_2_N, FeN, Fe_3_N, Fe_4_N) [5], occurred in the layer. The short-term (5–120 min) HTPGN at 510–560 °C (783–833 K) resulted in the formation of the nitrided layers of a thickness up to 12 μm [14]. Depending on the process parameters, the phase composition of the layers could be as follows: only expanded austenite (γ_N_) [14], CrN and Fe_4_N nitrides with expanded austenite (γ_N_) [14], or CrN nitrides, expanded austenite (γ_N_), and martensite (α’-Fe) [15]. The thickness of the nitrided layer could be still increased up to 72.2 μm by the long-term HTPGN process with the use of an active screen [12]. AISI 316L steel was also subjected to cathodic plasma electrolytic nitriding (CPEN) in the urea aqueous solution [16]. The obtained thickness of the nitrided layer, composed of expanded austenite (γ_N_) and FeN_0.076_ nitrides, was in the range of 13.82–28.06 μm.

Plasma gas nitriding was also used as one of the processes during various hybrid treatment of austenitic steels [7,13,17,18,19,20,21,22,23,24]. The simultaneous low-temperature plasma gas nitrocarburizing (LTPGNC) resulted in the formation of expanded austenite supersaturated with nitrogen and carbon (γ_NC_) in the surface layer [7] of the thickness of 10.5 μm. The thin Au coating (350 nm), pre-placed on the surface of 316 steel before the LTPGN with the use of an active screen [13], decreased thickness of the S-phase layer (1.9–3.4 μm) in comparison with the process without this coating. Austenitic 316L steel was also shot-peened before low-temperature plasma gas nitriding [17,18,19]. The thickness of the S-phase increased to 4.2 μm or to 5.7 μm, depending on the process parameters [17]. Shot peening could cause the formation of S-phase and nanocrystalline martensite (α’-Fe phase) of the increased thickness [18]. The surface of 316L steel was also shot-peened, low-temperature plasma gas carburized, and, finally, low-temperature plasma gas nitrided at the same temperatures for 2 h [19]. However, the thickness of such a hybrid layer didn’t exceed 14 μm. The other hybrid treatments of austenitic steels were as follows: selective laser melting (SLM) and LTPGN [20], cold spraying of 316L powder and LTPGN or LTPGC (low-temperature plasma gas carburizing), cold spraying, followed by LTPGC and LTPGN, or cold spraying, followed by a simultaneous LTPGNC process [21], laser metal deposition of 316L steel and nickel powders prior to LTPGN [22], plasma gas nitriding, followed by a multi-arc ion plating [23], or by physical vapor deposition (PVD) technique [24].

Low-temperature plasma gas carburizing (LTPGC) became a physical technique designed so as to obtain an advantageous combination of wear and corrosion behavior of austenitic stainless steels [25,26,27,28]. The temperature below 520 °C (793 K) resulted in the formation of the layer, which was composed of the austenite supersaturated with carbon (γ_C_) only [25,26,27,28]. Whereas the Cr_7_C_3_ chromium carbides, expanded austenite (γ_C_), and martensite (α’-Fe) appeared in the microstructure after plasma carburizing at higher temperature [25]. The plasma-carburized layers achieved the thickness up to 14 [26], 21 [28], 25 [27], or 50 μm [25]. The low-temperature plasma gas carburizing was also used in the hybrid treatment [21], obtaining the microstructure and thickness, as mentioned above.

Finally, the plasma boriding at diminished temperatures was also applied in order to improve the wear resistance of 316L austenitic steel [29,30,31]. Plasma gas boriding (PGB) resulted in the formation of the boride interlayer between the substrate, made of austenitic steel, and the thin nanostructured diamond film [29]. At 550 °C (823 K), only CrB borides were produced as a consequence of such a process. The boride layer consisted of the Fe_2_B phase if the process was carried out at 750 °C (1023 K). The further increase in process temperature up to 825 °C (1098 K) showed that the boride layer also included the CrB borides. During plasma paste boriding (PPB) of 316L steel [30], the surface of austenitic steel was coated with a paste containing 70% B_2_O_3_ (boron source) and 30% SiC (catalyst) in the form of a powder. Then, the process was carried out in the H_2_–Ar atmosphere at temperatures in the range of 700–800 °C (973–1073 K) for 3, 5, and 7 h under reduced pressure. In the microstructure, the two zones were identified with a predominant percentage of FeB and Fe_2_B iron borides, respectively. Besides, XRD revealed the chromium borides (CrB and Cr_2_B) as well as the nickel borides (Ni_3_B) in the borided layer. The maximal thickness of the layer was equal to 24 μm. If the samples were coated with the paste, consisting of only B_2_O_3_ powder, the thickness of the boride layers was reduced to a maximal value of 14 μm [31]. Whereas the phase composition was similar.

Despite the above-mentioned difficulties related to the activation of the steel surface, austenitic steels were also borided using the typical thermochemical treatment. Powder-pack boriding (P-PB), carried out in the range of temperature 800–950 °C (1073–1223 K), resulted in the formation of a boride layer without sacrificing corrosion resistance [33,34,35,36,37,38,39,40,41]. The two-phase boride layers (FeB + Fe_2_B) with strong zonation and smooth morphology (flat interface with the substrate) have been usually visible in the microstructure. The transition zone [33,35,36,39], also called diffusion zone [34,37,38,40,41], appeared between the boride layer and the substrate. Simultaneously, the chromium borides CrB [33,34,35,37,38,39,40,41] or Cr_2_B [34,35,37,38,40], nickel borides NiB [35,36], Ni_2_B [33,35,36,37,38], or Ni_3_B [34,38,39,40,41], and sometimes the manganese borides MnB [34] or Mn_2_B [38], as well as molybdenum borides (Mo_2_B [38]), were identified in the boride layer. The diffusion annealing was carried out after the P-PB process in order to eliminate the more brittle FeB phase [37]. The hybrid powder-pack processes were also applied, e.g., the boriding, followed by chromizing [41]. The thickness of boride layers, produced in austenitic steel by P-PB, usually didn’t exceed 60 μm [37,38,39,40,41]. AISI 316L steel was also subjected to boriding in liquid media [42,43], resulting in the formation of single-phase boride layers (only Fe_2_B) with a thickness of up to 40 [42] or 24 μm [43]. The typical low-temperature gas nitriding (LTGN) was carried out using 316L steel [44] as well as high velocity oxy-fuel (HVOF)-sprayed 316L coating [45] as a substrate material. The 6 μm thick nitrided layer consisted of S-phase (γ_N_) only [44,45]. The low-pressure LTGN process of HVOF-sprayed 316L steel was performed in ammonia gas (NH_3_) or in NH_3_ + H_2_ + N_2_ atmosphere [45]. The high-temperature gas nitriding (HTGN) was also carried out under reduced pressure prior to the laser powder bed fusion (L-PBF) process in order to increase the nitrogen concentration in the 316L steel powder [46] or as a solution nitriding [47]. However, the phase composition and the thicknesses of the nitrided layers weren’t provided by the authors. The nitriding of 316L steel was also carried out in liquid media using the special salt baths, consisting of potassium cyanide (KCN) and potassium cyanate (KCNO) [48] or cyanate and carbonate [49]. The nitrided layer consisted of the outer thin surface zone with Fe_3_O_4_ iron oxides, compound zone with nitrogen-expanded austenite (γ_N_), and the nitrides (Fe_4_N, Fe_2_N, CrN, Si_3_N_4_) of the thickness of 21 μm as well as diffusion zone with carbon-expanded austenite (γ_C_) [48]. Powder-pack carburizing with coconut shell and rice husk, as well as coconut shell charcoal as a carbon source, was tried to use in order to produce wear- and corrosion-resistant layers in 316L steel [50,51]. However, their microstructure wasn’t described in detail. The source of carbon wasn’t provided in the case of low-temperature carburizing of 316L steel [52]. Apart from the above-mentioned technological superficial layers, usually called “layers” and produced using non-decremental techniques, the coatings were also produced using incremental techniques in order to improve the wear resistance of 316L steel. Titanium nitride (TiN) coatings with a thickness of 1.4 [53] and 1.6–2.4 μm [54] were deposited by physical vapor deposition (PVD).

In recent years, alternative surface treatments were proposed in order to increase the case depth of the surface layers. Such processes as laser heat treatment (LHT), laser surface alloying (LSA), or laser cladding (LC) were intensively developed for a wide range of applications [55,56]. LHT processes, carried out with or without re-melting of the steel substrate, could produce the hardened surface layer in constructional [57,58] or tool steels [58,59] as a consequence of laser quenching. Laser processing technology also promoted the superficial incorporation of hard phases into the metals and their alloys by LC or LSA. The conditions of such processes could be selected this way that they minimized (LC) or promoted (LSA) the dissolution of these hard phases in the re-melted zone. It provided the coatings (by LC) or superficial surface layers (by LSA) with a wide diversity of microstructures and properties [32,60].

Laser surface alloying with boron (as an alloying material) was intensively developed for constructional or tool steels [58,61,62], nodular cast iron [63], titanium and its alloys [64,65,66,67], or Ni-based alloys [68,69]. Such a process was often called laser boriding [32]. In the case of austenitic stainless steels, LSA processes were initially applied in order to improve the surface hardness and wear resistance by incorporating carbides [70,71,72]. Next, investigations concerned the LSA of austenitic steels in which boron was used as one of the alloying elements [73,74]. A mixture of molybdenum and boron powders became an alloying material to coat the surface of Sandvik 1802 steel [73]. As a consequence of laser re-melting, the hard borides appeared in the microstructure, increasing the surface hardness. The thermal sprayed coatings were also used as an alloying material. The powder of NiCoCrB-alloy with about 1 wt% of boron was pre-placed on the surface of 316L steel using flame spraying [74]. As a consequence, the hard ceramic phases (borides and boro-carbides) appeared in the sprayed coating. The next step consisted of laser re-melting. The increase in hardness up to 410 HV, as well as the improved resistance to cavitation corrosion, was observed. Finally, the LSA of 316L steel with only boron was studied [75]. The significant increase in hardness and wear resistance characterized such a laser-alloyed layer when compared to the untreated 316L steel. However, the corrosion resistance, being an important property of the surface layer produced on the austenitic 316L steel, wasn’t studied in this paper.

In the present study, the laser surface alloying with boron and selected metallic elements was investigated in order to improve the wear behavior of austenitic 316L steel without sacrificing its corrosion resistance. The surface layers were produced using alloying materials in the form of powders as follows: boron, boron and nickel, boron and mixture of nickel and chromium, as well as boron and Stellite-6. In the present work, only the effects of alloying materials on the microstructure and cohesion of laser-alloyed layers were analyzed. The properties of the laser-alloyed layers, such as microhardness or wear and corrosion resistance, would be described in the next paper.

## 2. Materials and Methods

### 2.1. Materials and Specimens

**Figure 1:**
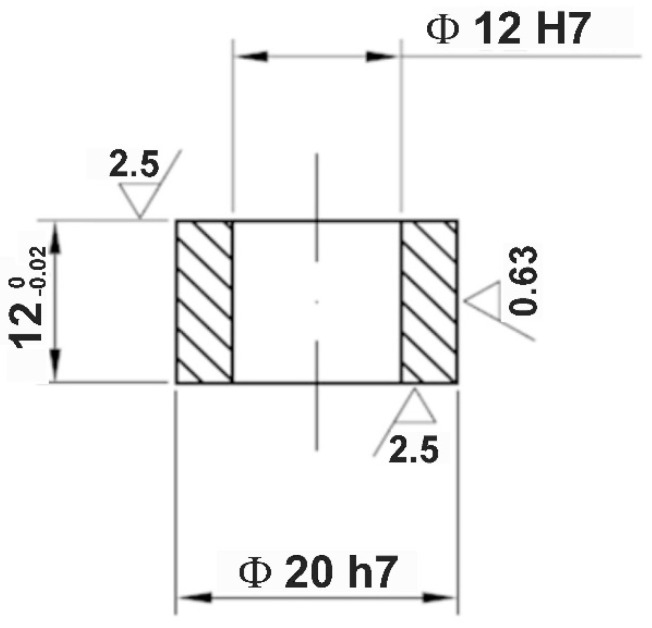
Shape and dimensions of the specimens used.

AISI 316L austenitic stainless steel was used as the alloyed material, i.e., substrate material. Its chemical composition, provided by the supplier of the material, is presented in Table 1. Specimens in the shape of a ring with an external diameter of 20 mm, internal diameter 12 mm, and height 12 mm were prepared by machining. The outer surface was formed by finish turning. The dimensions of the specimens are shown in Figure 1 in detail.

The alloying materials were prepared using the powders of amorphous boron B with purity ≥95% and particle size ≤1 μm (Sigma Aldrich, Inc., Poznan, Poland), nickel Ni with purity ≥99.7% and particle size ≤50 μm (Sigma Aldrich, Inc., Poznan, Poland), a mixture of nickel and chromium Ni–Cr with mass ratio 4:1 and particle size ≤25 μm (Euromat, Wroclaw, Poland), as well as Stellite-6 alloy with particle size 25–53 μm (Euromat, Wroclaw, Poland). The chemical composition of Stellite-6 powder is specified in Table 2. Cobalt was the element of the highest concentration in this powder.

### 2.2. Laser Surface Alloying of 316L Steel

LSA processes were carried out by the re-melting technique. It was a two-stage process. The first step consisted of the prior deposition of coating with the alloying material onto the alloyed material (substrate). The subsequent re-melting of this coating, together with the surface of the alloyed material, provided the laser-alloyed layer [32]. The appropriate powders, as mentioned above, were blended with a diluted polyvinyl alcohol solution. The prepared paste was deposited on the outer cylindrical surface of the specimens made of 316L steel. The thickness of all the coatings with alloying material was equal to 200 μm. It was measured by the thickness gauge of coatings Positector 6000 (DeFelsko, Poznan, Poland) using the phenomenon of the magnetic induction and eddy currents. The following powders were used as an alloying material:

- amorphous boron,
- mixture of amorphous boron and Stellite-6 powders with mass ratio 1:1,
- mixture of amorphous boron and nickel powders with mass ratio 1:1,
- mixture of amorphous boron and Ni–Cr powders with mass ratio 1:1.

Such alloying materials weren’t selected by chance. The LSA with boron resulted in the formation of hard ceramic phases (iron, chromium, and nickel borides) in the austenitic matrix. It was the reason for the improved hardness and tribological properties of 316L steel [75]. It was expected that the mixtures of boron and selected elements could diminish the laser beam power during LSA. The melting points of Ni, Cr, or Co are lower than that of boron. Besides, the formation of chromium and nickel borides could cause the diminished concentration of these elements in the austenitic matrix and, as a consequence, the worsened corrosion resistance. The presence of Ni, Cr, or Co in alloying material could partially enrich the austenitic matrix by these elements.

**Figure 2:**
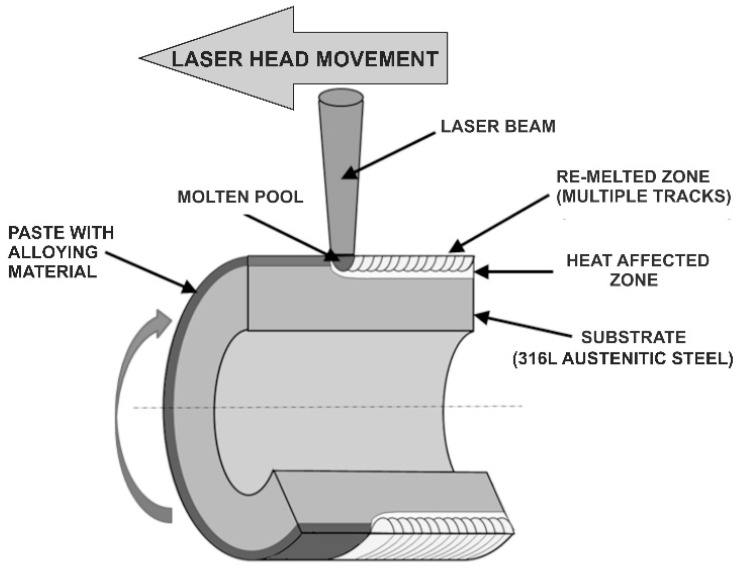
Two-step technique of laser surface alloying (LSA) by re-melting.

During the second stage of LSA, the samples prepared by this way were subjected to laser treatment. As a consequence, the alloying material was re-melted together with the substrate (alloyed material). The course of the LSA by re-melting is shown in Figure 2. The aim of the use of boron as alloying material was to produce the hard metal borides in the surface layer in order to improve tribological properties of austenitic steel without a substantial decrease in its corrosion resistance.

**Figure 3:**
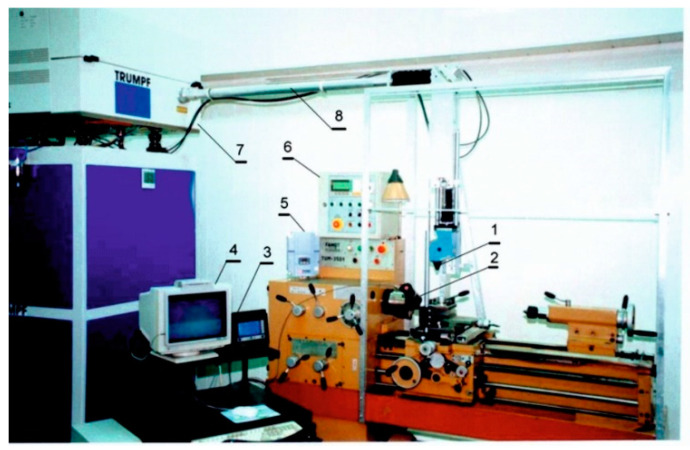
The equipment used for laser surface alloying of 316L steel: 1—focusing head, 2—temperature measuring head (Raytek Thermalert, Poznan, Poland), 3—temperature measuring system (Raytek Thermalert), 4—computer temperature recording, 5—frequency converter for the stepless regulation of rotational speed, 6—outer controller of the laser beam, 7—resonator TLF 2600t, 8—outer optical system.

LSA processes of 316L steel were carried out using the appropriate workstation. Its main part was composed of a continuous CO_2_ laser TLF 2600 Turbo (TRUMPF, Poznan, Poland) of the nominal power 2.6 kW, coupled with a turning lathe that enabled rotation of the specimens and feed motion of the focusing head. All the equipment used for laser surface alloying is shown in Figure 3. The TEM_01_ multiple mode was characteristic of the laser beam applied. It was a special mode, generated by a superposition of the two TEM_01_ modes, rotated 90° with respect to one another. As a consequence, the toroidal profile of irradiance was obtained. Such a shape of the irradiance profile showed that the effects of laser beam activity didn’t depend on the direction of movement of the laser beam in relation to the treated surface. The focusing mirror was characterized by curvature 250 mm, diameter 48 mm, and focal length 125 mm. The distance from the bottom edge of a fixing holder of focusing mirror to the surface of the paste coating with alloying material was equal to 106.8 mm. Such a distance was longer than the position of the focused beam, located in the distance of 91.8 mm from the bottom edge of a fixing holder of the mirror. It resulted in the laser beam diameter *d* = 2 mm.

Laser surface alloying was carried out in argon shielding at a pressure of 0.2 MPa, measured by the pressure-reducing valve installed on the cylinder with argon. Argon of high-purity (6.0) was supplied outside the laser beam through the same coaxial nozzle in order to protect the alloyed surface against oxidation.

**Figure 4:**
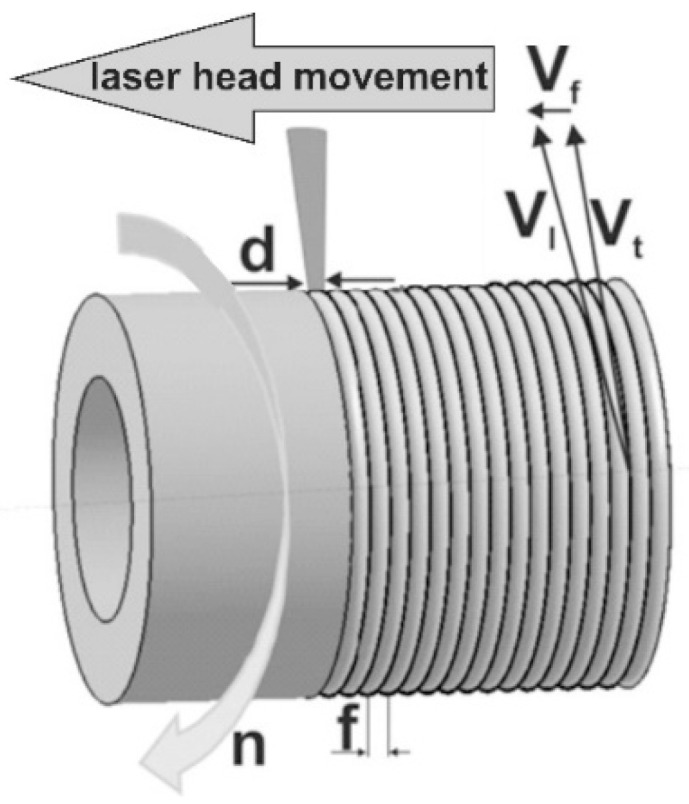
The technique of multiple tracks’ formation along the helical line; *d*—laser beam diameter; *v*_f_—the rate of feed; *v*_l_—scanning rate; *v*_t_—tangential speed; *n*—rotational speed; *f*—distance from track to track.

The laser tracks were arranged as multiple tracks. They were formed along the helical line on the outer cylindrical surface of the specimens (Figure 4). The parameters of relative motion of focusing laser head with regard to the alloyed surface were as follows: scanning rate *v*_l_ = 2.88 m·min^−1^, and the distance between the axes of adjacent tracks *f* = 0.28 mm. The value of *f* resulted directly from the feed rate *v*_f_, which was equal to 0.28 mm per revolution. Whereas scanning rate *v*_l_ was calculated based on the rotational speed of the specimen (*n* = 45.85 min^−^^1^), the external diameter of the specimen (*D* = 20 mm), and feed rate (*v*_f_ = 0.28 mm per revolution). The way of calculations is shown below.

The angular speed of the specimen *ω* could be expressed as:

(1) $$\omega =2\times \pi \times n\;\left(min^{-1}\right)$$

where *n* is rotational speed (min^−1^).

The tangential speed of the specimen *v*_t_ was equal to 2.88 m·min^−1^ and depended on its external diameter and angular speed:

(2) $$v_{t}=\omega \times \frac{D}{2}=2\times \pi \times n\times \frac{D}{2}=2.88\;\left(m\;\cdot \;min^{-1}\right)$$

where *ω* is the angular speed (min^−1^), *D* is an external diameter of the specimen (m).

Feed rate *v*_f_ (0.28 mm per revolution) could be expressed in m·min^−1^ according to the relationship:

(3) $$v_{f}=0.00028\times n=0.01284\;\left(m\;\cdot \;min^{-1}\right)$$

where *n* is rotational speed (min^−1^).

According to Figure 4, the scanning rate *v*_l_ could be calculated as a resultant value of tangential speed and feed rate:

(4) $$v_{l}=\sqrt{v_{t}^{2}+v_{f}^{2}}\approx 2.88\;\left(m\;\cdot \;min^{-1}\right)$$

where *v*_t_ is the tangential speed (m·min^−1^), *v*_f_ is the feed rate (m·min^−1^).

The result of the calculation, according to Equation (4), indicated that the scanning rate (*v*_l_) mainly depended on the tangential speed (*v*_t_), i.e., on the rotational speed of the sample during laser processing.

Based on the laser beam diameter (*d*) and the distance between the axes of adjacent tracks (*f*), the overlapping of laser tracks (*O*) could be calculated as follows:

(5) $$O=\frac{d-f}{d}\times 100\%=86\%$$

**Figure 5:**
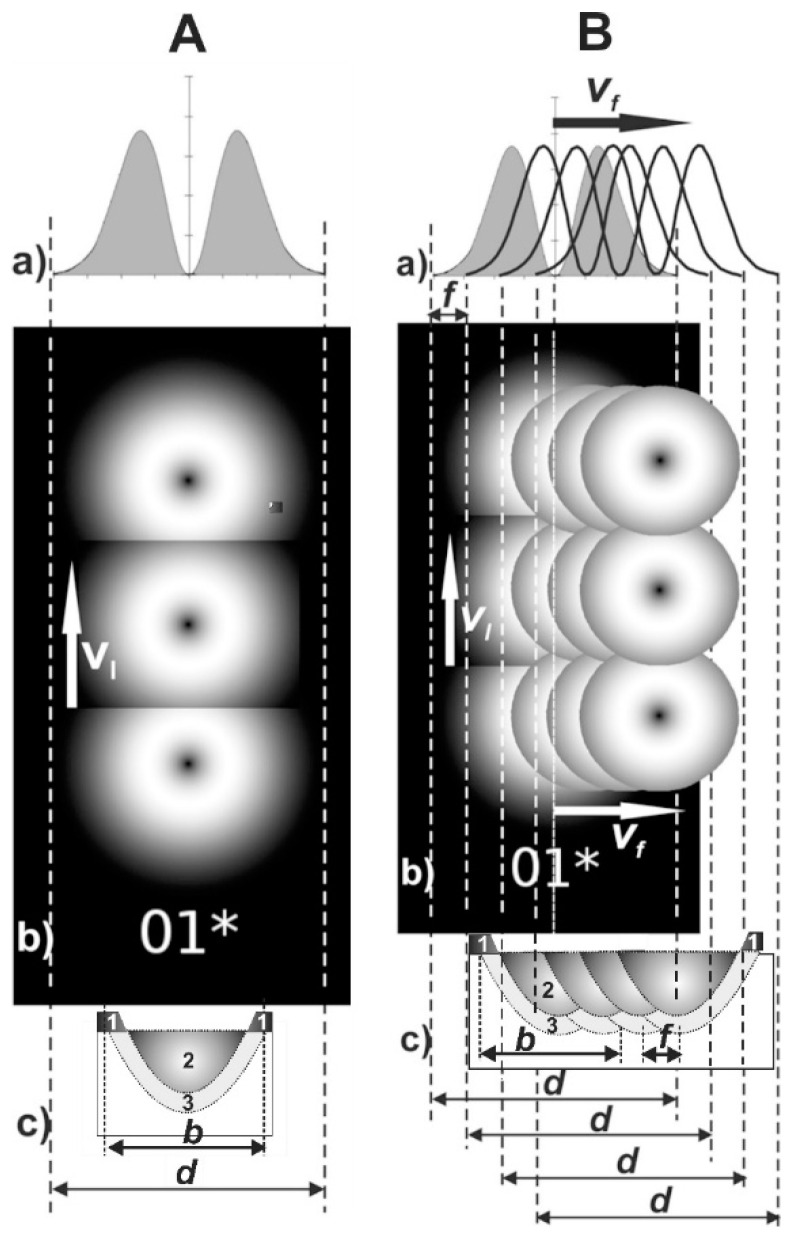
Irradiance profiles and their influence on the dimensions of single (**A**) and multiple (**B**) laser tracks produced during LSA by re-melting: radial profile of irradiance (**a**), projection of the irradiance profile onto the laser-alloyed surface (**b**), the effect of irradiance profile on the dimensions of the produced laser tracks (**c**); *d*—laser beam diameter, *v*_f_—feed rate, *v*_l_—scanning rate, *b*—width of laser track, *f*—distance from track to track, 1—paste coating with alloying material, 2—re-melted zone, 3—heat-affected zone.

In Figure 5, the irradiance profile and its general effect on the dimensions and microstructure of both single laser track and multiple laser tracks are shown. A radial profile of the irradiance is visible in Figure 5(Aa,Ba). The projection of the irradiance profile onto the laser-alloyed surface (Figure 5(Ac,Bc)) and the influence of the irradiance profile on the microstructure and dimensions of the produced laser tracks (Figure 5(Ac,Bc)) are also presented. The microstructure of the surface layer after LSA usually consisted of re-melted (laser-alloyed) zone (2) and heat-affected zone (3). On the contrary to laser cladding (LC), LSA didn’t change the dimensions of the treated object substantially [32].

The changeable laser beam power *P* was used during LSA processes. Laser surface alloying with boron only was carried out at the laser beam power of 1.82 kW. In the case of other alloying materials, i.e., mixtures of boron and various metallic elements, the two values of *P* were applied: 1.43 W and 1.56 kW. All the parameters of LSA processes are shown in Table 3, providing the type of coating with alloying material and its thickness (*t*_C_), laser beam diameter (*d*), scanning rate (*v*_l_), overlapping (*O*), as well as laser beam power (*P*) and corresponding averaging irradiance (*E*). The averaging irradiance was calculated this way that the laser beam power had been divided by the circular surface of the laser beam. Such parameters were selected based on the experience of authors in laser surface alloying [58,61,62,66,67,68,69,75] and a certain number of tests.

### 2.3. Microstructure Observations

After the LSA processes described above, the ring-shaped specimens were cut out perpendicular to the treated surface, across the laser tracks produced. Cross-sections of the laser-alloyed samples were prepared for microstructure observations in the plane perpendicular to the scanning direction. The metallographic specimens were done this way that at first, the cut samples were mounted in a conductive resin. Next, they were polished using the abrasive paper of different granularity. Finally, the Al_2_O_3_ slurry was applied during polishing. In order to reveal the microstructure, the laser-alloyed specimens were etched with a special reagent, which was prepared using the mixture of FeCl_3_ (25 g), HCl (25 mL), and H_2_O (100 mL). The revealed microstructures were observed with the use of an optical microscope (OM) Metaval (Carl Zeiss, Poznan, Poland) and scanning electron microscope (SEM) Vega 5135 (TESCAN, Poznan, Poland).

### 2.4. X-ray Microanalysis and Phase Analysis

The PGT Avalon X-ray microanalyzer (Princeton Gamma Tech, Poznan, Poland) equipped with EDS was used in order to measure the concentrations of selected elements in the laser-alloyed layer using a 55° take-off angle. The contents of iron, chromium, nickel, molybdenum, cobalt, and boron were measured. Such elements corresponded to the most important elements of 316L steel (Fe, Cr, Ni, Mo), as well as to the elements, which were the main components of alloying materials (B, Ni, Cr, Co).

The accelerating voltage was equal to only 12 kV. It enabled more accurate measurements of such a light element as boron. Si (Li) detector with an ultra-thin window was applied. The standardless quantitative analysis and ZAF (atomic number-absorption-fluorescence) matrix correction algorithms for SEM bulk analysis were used. However, a special procedure was used in the case of boron measurements. The aim of this procedure was to eliminate the contamination by carbon. Hence, the calibration of the equipment was carried out using the real standards of boron contents. FeB and Fe_2_B phases, produced on the borided Armco iron, were used as the standards. Carbon did not dissolve in iron borides. The standards were prepared using the same procedure as for samples. According to the Fe–B equilibrium system [76], the boron concentrations in the iron borides should correspond to 16.23 wt% B in FeB and 8.83 wt% B in Fe_2_B. The PANalytical EMPYREAN X-ray diffractometer (Malvern Panalytical Ltd., Poznan, Poland) with Cu K_α_ radiation was used during the phase analysis of all the laser-alloyed layers. Based on the X-ray diffraction (XRD) patterns obtained, the corresponding phases were identified.

### 2.5. Cohesion

The study of the cohesion of laser-alloyed layers consisted of conducting the typical test, usually used to study the adhesion of coatings. Rockwell C indentation test was employed to evaluate the cohesion of the surface layers produced. The test was performed this way that the cone diamond tip was pressed into the material surface, as in the case of the classic hardness test by this method. The condition necessary to meet was that the thickness of the sample had to exceed at least ten times the depth of the indent obtained. As a result of the test, there was usually the formation of cracks in the surface layer and plastic deformation within the substrate.

**Figure 6:**
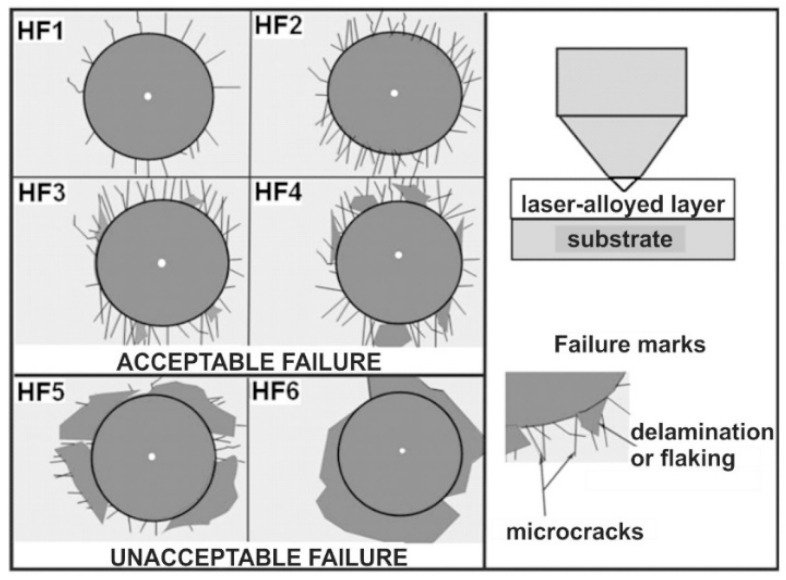
OM microstructure of laser-borided layer, produced on austenitic 316L steel with a dilution ratio of 0.54 using laser beam power *P* = 1.82 kW; 1—re-melted zone (MZ), i.e., laser-alloyed layer, 2—substrate material (316L steel).

Determination of the degree of layer cohesion was carried out based on the observation of the indents using an optical microscope (OM) Metaval (Carl Zeiss, Poznan, Poland). Attention was paid to the resulting microcracks and possible signs of flaking or delamination of the material around the indent. The observed test effects were compared with the scale of the patterns described in the VDI (Verein Deutscher Ingenieure Normen) 3198 standard [77]. This standard classified the observed surface destruction according to six HF1–HF6 patterns. The scale of these patterns is shown in Figure 6. The four patterns (HF1–HF4) corresponded to an acceptable layer cohesion. On the other hand, the two patterns (HF5 and HF6) showed damage around the indent, indicating insufficient cohesion.

## 3. Results and Discussion

### 3.1. Selection of the Laser Surface Alloying Parameters

The main parameters of LSA of 316L austenitic stainless steel, such as the type of alloying material and its thickness (*t*_C_), laser beam diameter (*d*), scanning rate (*v*_l_), overlapping (*O*), as well as laser beam power (*P*) and corresponding averaging irradiance (*E*), strongly influenced the quality and properties of the produced surface layers. Appropriately selected parameters of laser processing resulted in obtaining the homogeneous microstructure in the re-melted zone and the uniform thickness of the formed layers [75] because of the relatively high overlapping. In the present study, the parameters of laser surface alloying of 316L steel with boron as well as with boron and some metallic elements were selected based on the previous investigation and authors’ experience in the design of LSA processes.

Laser surface alloying of austenitic steel 316L using various alloying materials was described in papers [72,74,78,79]. The laser processing parameters used differed significantly. The alloying material consisted of one or more types of powders and sometimes contained boron or boron compounds. A laser beam power (*P*) in the wide range of 0.595–3 kW, a laser beam diameter (*d*) from 0.95 to 3.5 mm, and a scanning rate (*v*_l_) from 5 to 20 mm·s^−1^ were used. The overlapping (*O*) was in the range of 50–70%. The thickness of the preplaced coating with alloying material (*t*_C_) ranged from 190 to 250 μm [74] or was equal to 750 μm [79]. Sometimes, this coating was characterized in mg·cm^−2^, ranging from 2.5 to 20 mg·cm^−2^ [72,78].

The LSA of 316L steel exclusively with boron was the first time studied in the paper [75]. The laser beam powers of 1.43, 1.56, and 1.82 kW were used. The thickness of the preplaced coating with amorphous boron was equal to 200, 215, and 230 μm, according to the increasing beam power. The other laser processing parameters were as follows: laser beam diameter *d* = 2 mm, scanning rate *v*_l_ = 2.88 m·min^−^^1^, and overlapping *O* = 86%. This study [75] confirmed that the dilution ratio (*DR*) strongly influenced the quality of the laser-alloyed layers with boron, produced on 316L steel. The minimal dilution ratio (*DR* = 0.37) was indicated, which caused the formation of laser-borided layers without defects, such as microcracks and gas pores. Such a good quality of the surface layer produced was obtained at a laser beam power of 1.82 kW using the coating with boron of thickness equal to 230 μm. The use of laser beam power of 1.43 or 1.56 kW resulted in the formation of defected laser-alloyed layers with unacceptable quality. Therefore, the dilution ratio was adequately selected for all the produced laser-alloyed layers in this study and calculated according to the equation:

(6) $$DR=1-\frac{t_{C}}{d_{\mathrm{MZ}}}$$

where *t_C_* is the thickness of paste coating with alloying material (μm), *d*_MZ_ is the averaging depth of the re-melted (laser-alloyed) zone (μm).

The LSA with boron only (i.e., laser boriding) in the present study was carried out with similar parameters as in the paper [75]. However, the thickness of the preplaced coating with boron was diminished to 200 μm in order to increase the dilution ratio above the minimum required value (0.37). Hence, only one laser beam power (1.82 kW) was used in this case.

The alloying material was also modified by the use of selected metallic elements. The melting points of such materials as Stellite-6 (1285 °C), nickel (1453 °C), or chromium (1857 °C) were lower than that of boron (2076 °C). It was expected that the use of powder mixtures of boron and selected metallic elements as alloying materials, instead of only boron powder, would make it possible to reduce the laser beam power needed to produce the laser-alloyed layers of acceptable quality. Hence, the thickness of the preplaced coating was the same for all the alloying materials (200 μm), and the laser beam power used was diminished to 1.43 or 1.56 kW.

The results of the calculations are shown in Table 4. All the laser-alloyed layers were produced at the dilution ratio (*DR*) above 0.37. In the case of a laser-alloyed layer with only boron, the highest *DR* value of 0.54 was obtained at the laser beam power *P* = 1.82 kW. However, the use of mixtures of boron and selected metallic elements as alloying material allowed to use the lower laser beam powers (1.43 or 1.56 kW) in order to produce the laser-alloyed layers without defects in the form of microcracks or gas pores. It was caused by the diminished melting points of the coatings with mixtures of boron and metallic elements. Their dilution ratio ranged from 0.41 to 0.43 for laser beam power of 1.43 kW and from 0.48 to 0.49 in the case of the higher value of *P* (1.56 kW).

### 3.2. Microstructure of Laser-Alloyed Layer with Boron

**Figure 7:**
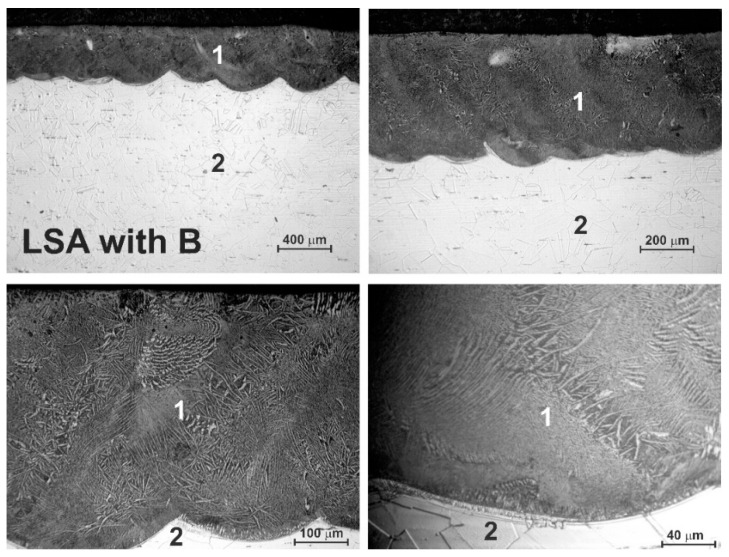
OM microstructure of laser-borided layer, produced on austenitic 316L steel with a dilution ratio of 0.54 using laser beam power *P* = 1.82 kW; 1—re-melted zone (MZ), i.e., laser-alloyed layer, 2—substrate material (316L steel).

The OM microstructure of the laser-alloyed layer with boron only is shown in Figure 7. The produced composite surface layer was free from the microcracks or gas pores due to the relatively high dilution ratio (*DR* = 0.54). Only the two zones were visible in the laser-alloyed 316L steel: MZ - re-melted zone (1) and the substrate material (2). The differences in the microstructure of the heat-affected zone (HAZ) and the substrate weren’t visible because of the extended stability of austenite up to room temperature and no possibility to change this structure by martensite transformation during fast cooling. Therefore, the laser-alloyed layer with boron was composed of the only re-melted zone (MZ). The thickness of the laser-alloyed layer with boron was uniform. It resulted from the relatively high overlapping used (86%). The averaging depth of the MZ, i.e., laser-alloyed layer, was equal to 432 μm. Based on the high concentrations of alloying elements in 316L steel, it was expected that the microstructure of the laser-alloyed layer with boron would be composed of iron, chromium, and nickel borides in an austenitic matrix.

**Figure 8:**
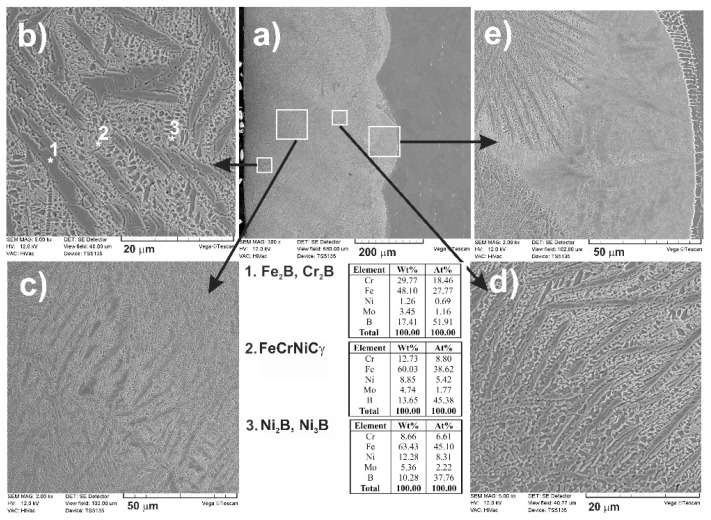
SEM microstructure of laser-borided layer, produced on austenitic 316L steel with a dilution ratio of 0.54 using laser beam power *P* = 1.82 kW; the entire re-melted zone (**a**), areas close to the surface with Fe_2_B and Cr_2_B borides in the form of polygons or needle-shaped sticks as well as darker and finer Ni_2_B or Ni_3_B phases (**b**) and (**c**), area of needle-shaped borides (**d**), end of the re-melted zone (**e**); 1—Fe_2_B or Cr_2_B borides, 2—austenitic matrix (FeCrNiCγ), 3—Ni_2_B or Ni_3_B borides.

A detailed analysis of the formed microstructure was conducted using a scanning electron microscope (Figure 8). SEM images confirmed the composite microstructure of a laser-alloyed layer. From a cross-section of the laser-alloyed layer with boron, formed on austenitic 316L steel (Figure 8a), some areas differing in the microstructure were distinguished (Figure 8b–d). They were shown at higher magnification and characterized. The presence of possible phases was suggested by the energy-dispersive X-ray spectroscopy (EDS or EDX) point analysis for the 1, 2, and 3 locations. In the areas close to the surface (Figure 8b,c), the iron and chromium borides (Fe_2_B and Cr_2_B) appeared in the form of polygons or needle-shaped sticks (location 1). The areas with austenitic matrix (FeCrNiCγ) as well as the nickel borides (Ni_2_B or Ni_3_B) in the form of darker and finer precipitates were also observed, probably in locations 2 and 3, respectively. The segregation of boron during resolidification caused diversification in the microstructure of MZ with respect to both percentage of iron, chromium, or nickel borides and their form.

The literature data reported the presence of FeB and Fe_2_B phases with strong zonation as well as chromium and nickel borides in austenitic steels after diffusion boriding [30,31,32,33,34,35,36,37,38,39,40,41]. After laser surface alloying with boron (i.e., laser boriding), the same hard ceramic phases were expected in MZ. However, the LSA with boron resulted in producing the composite layer consisting of hard borides in a soft austenitic matrix [75]. Sometimes, the alloyed boro-carbides occurred in such a layer, e.g., in laser-fabricated Fe–Ni–Co–Cr–B austenitic alloy on 316L steel [74]. A small amount of ferrite δ was often detected in the MZ [80]. Usually, the precipitation of boro-carbides was observed at a low cooling rate, which also eliminated ferrite δ from the microstructure, at least up to the depth penetrated by Cu K_α_ radiation [75].

**Figure 9:**
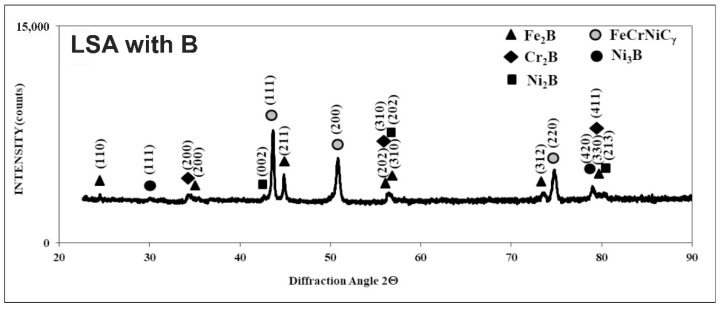
XRD patterns of laser-borided layer, produced on austenitic 316L steel with a dilution ratio of 0.54 using laser beam power *P* = 1.82 kW.

Directly after LSA, the treated surface was subjected to the phase analysis by XRD. Cu K_α_ radiation was used for all the laser-alloyed layers in order to obtain the XRD patterns. According to the literature data, such radiation penetrated the surface of metals and their alloys to a relatively low depth [81,82,83]. Probably, the penetration depth was not more than tens of micrometers for the surface layers of metallic materials. Phase analysis by XRD (Figure 9) confirmed the presence of Fe_2_B iron borides, Cr_2_B chromium borides, Ni_2_B and Ni_3_B nickel borides, as well as an austenitic matrix (FeCrNiCγ) in a laser-alloyed layer (re-melted zone) with boron. The absence of the oxides indicated sufficient argon protection during laser processing. Some peaks from chromium and nickel borides coincided with the peaks from iron borides. Hence, the presence of compound (Fe,Cr)_2_B and (Fe,Ni)_2_B borides was possible. The intensities of the peaks from hard borides were diminished when compared to the laser-alloyed layer, produced at a lower dilution ratio (*DR* = 0.37) and reported in the previous paper [75]. Simultaneously, the intensity of the peaks from austenite (FeCrNiCγ) was increased. It resulted from the higher value of *DR* (0.54) applied in the present study. In spite of the relatively low cooling rate, such a high dilution ratio was the reason for the absence of M_23_(C,B)_6_ boro-carbides (where M = Fe, Cr) in the laser-alloyed layer with boron. The peaks from ferrite δ weren’t identified in MZ because of this low cooling rate, at least up to a depth, penetrated using Cu K_α_ radiation [75]. The presence of alloyed boro-carbides with high chromium concentration was confirmed in the case of the laser-borided layer with a dilution ratio of 0.37 [75]. It could cause worse corrosion behavior because of the diminished chromium content in the austenitic matrix.

**Figure 10:**
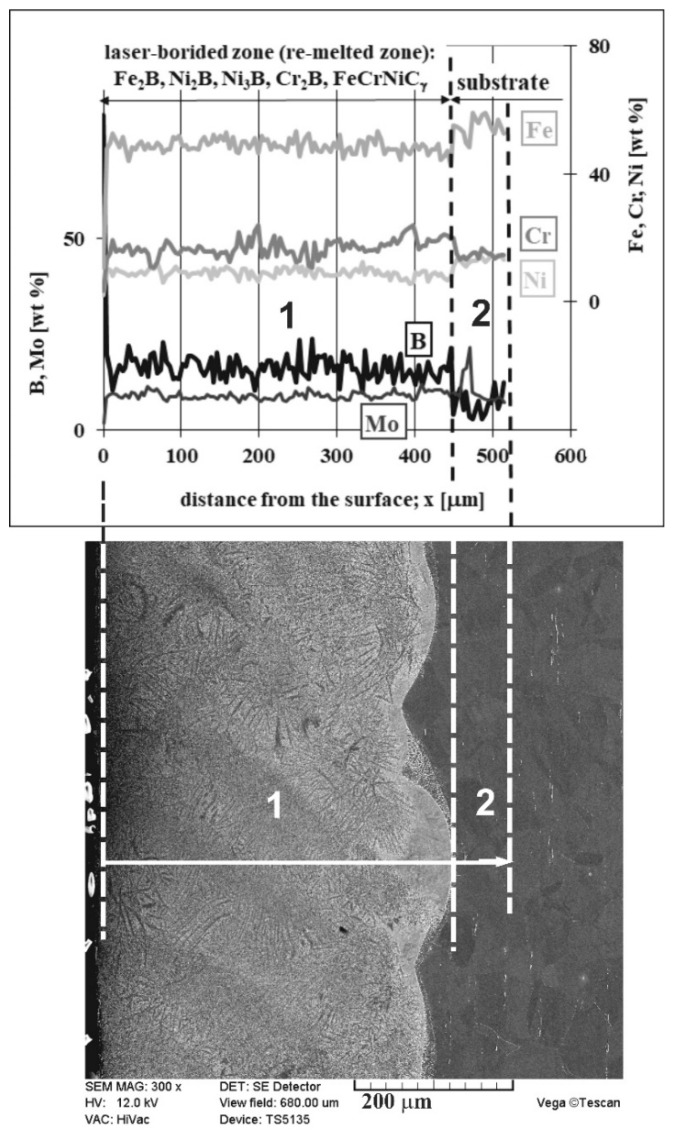
Results of linear X-ray microanalysis (EDS) of laser-borided layer, produced on austenitic 316L steel with a dilution ratio of 0.54 using laser beam power *P* = 1.82 kW.

The composite microstructure with hard ceramic phases, such as borides in the austenitic matrix, changed the chemical composition of the laser-borided layer. Hence, the profiles of the main elements in this surface layer were analyzed by the EDS method. The linear X-ray microanalysis was conducted, taking into account boron concentration (used as alloying material) as well as iron, chromium, nickel, and molybdenum contents (the primary elements of 316L steel). The SEM image of the microstructure, the line of measurements, and the linear profiles of the considered elements are shown in Figure 10. Such measurements provided the data on the chemical composition across the laser-borided layer. In general, the locations of the separate measurements were random. Although the use of the relatively low accelerating voltage (12 kV) limited the volume of measuring spots, they included the multiphase microstructure. However, based on the XRD patterns and the measured concentration profiles of elements, the conclusions could be formulated regarding the probable fraction of the phases occurring in MZ, i.e., laser-alloyed layer.

Compared to the substrate material, the averaging concentrations of nickel and iron in the laser re-melted zone were significantly reduced. In general, the increase in averaging contents of boron, which was used as an alloying material, was the reason for such a situation. In the presence of boron, iron and nickel demonstrated the tendency of creating the iron or nickel borides. Such a situation had to diminish the averaging concentrations of iron and nickel. It also confirmed the appreciable percentage of iron and nickel borides (Fe_2_B, Ni_2_B, and Ni_3_B) in the laser-alloyed layer. Usually, the relatively high boron concentrations were accompanied by relatively low iron and nickel contents. Probably, such results indicated the significant percentage of iron or nickel borides in measuring spots. Whereas the relatively high contents of iron and nickel corresponded to the lower boron concentrations, indicating the greater percentage of the austenitic matrix. Such a good correlation regarding the profiles of iron and nickel contents could confirm the presence of complex (Fe,Ni)_2_B borides in some areas. Some peaks of Fe_2_B and Ni_2_B phases overlapped in XRD patterns (Figure 9).

The concentration profiles of iron and chromium were also characterized by a good correlation. Hence, the (Fe,Cr)_2_B phase could also appear in MZ. Based on the XRD patterns (Figure 8), the presence of these complex borides could be possible. Some peaks of Fe_2_B and Cr_2_B phases overlapped. However, the limited tendency to form the chromium borides caused an increase in the averaging chromium contents in the laser-alloyed layer compared to the substrate. It indicated the relatively low fraction of chromium borides in MZ. The probable spots with relatively high percentages of chromium borides corresponded to the lower chromium contents, which were accompanied by high boron concentrations. It meant that the appreciable amount of chromium remained in the austenitic matrix. Hence, the spots with higher chromium concentrations indicated the presence of austenite. The reduced percentage of the matrix (austenite) in MZ due to the formation of borides was the reason for increasing the mean content of chromium in this region.

In general, the concentration profile of molybdenum in MZ didn’t differ from the profile below this zone. It could result from the limited tendency to form molybdenum borides. The unexpected increase in the molybdenum concentration right below the re-melted zone seemed to be random.

### 3.3. Microstructure of Laser-Alloyed Layer with Boron and Stellite-6

Laser surface alloying of 316L steel with a mixture of B and Stellite-6 powders with mass ratio 1:1 was carried out at laser beam powers of 1.43 and 1.56 kW. The thickness of the preplaced coating with alloying material was equal to 200 μm. After the treatment using the lower laser beam power (1.43 kW), the averaging thickness of the re-melted zone *d*_MZ_ = 338 μm was measured. Hence, the dilution ratio *DR* = 0.41 was obtained. In the case of applying the laser beam power of 1.56 kW, the values of *d*_MZ_ and *DR* were equal to 384 µm and 0.48, respectively. Other laser processing parameters were the same in both cases, i.e., laser beam diameter *d* = 2 mm, scanning rate *v*_l_ = 2.88 m·min^−1^, and overlapping *O* = 86%.

**Figure 11:**
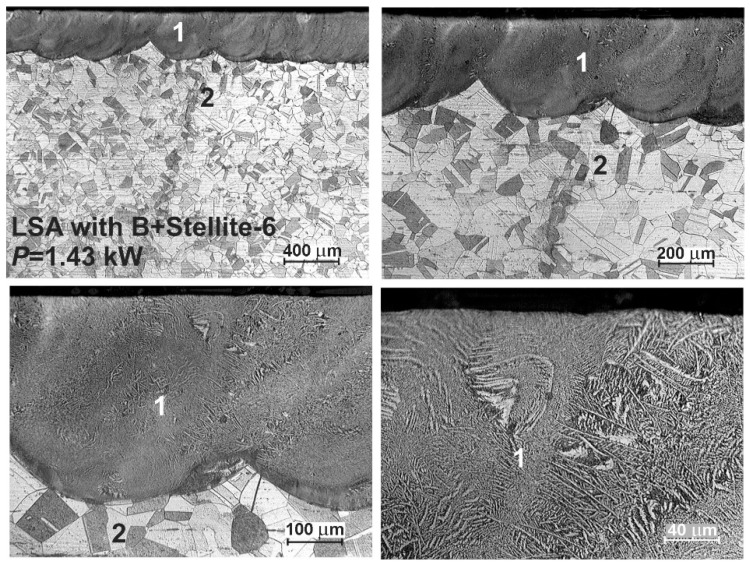
OM microstructure of laser-alloyed layer with boron and Stellite-6, produced on austenitic 316L steel with a dilution ratio of 0.41 using laser beam power *P* = 1.43 kW; 1—re-melted zone (MZ), i.e., laser-alloyed layer, 2—substrate material (316L steel).

**Figure 12:**
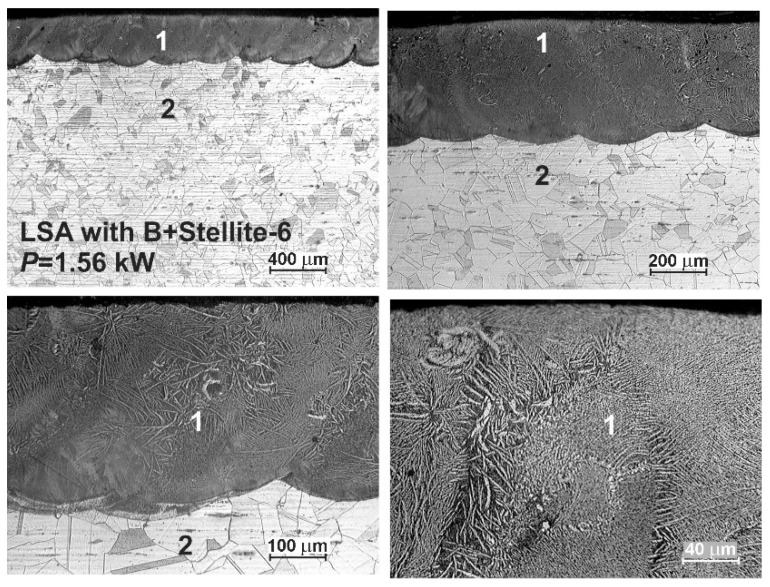
OM microstructure of laser-alloyed layer with boron and Stellite-6, produced on austenitic 316L steel with a dilution ratio of 0.48 using laser beam power *P* = 1.56 kW; 1—re-melted zone (MZ), i.e., laser-alloyed layer, 2—substrate material (316L steel).

The OM microstructures of the laser-alloyed layers with boron and Stellite-6 are shown in Figure 11 and Figure 12. Like in the previous case, the presence of re-melted zone (1) and austenitic substrate material (2) was confirmed, and the laser-alloyed layer was composed of only MZ. The relatively uniform thickness was characteristic of this re-melted zone due to applying the high overlapping (86%). The produced laser-alloyed layer revealed composite nature, consisting of hard borides in the austenitic matrix, and obtained a high quality without defects, such as microcracks or gas pores. Like previously, the effects of laser irradiation weren’t observed in the heat-affected zone due to no possibility to change the austenitic structure by martensite transformation during fast cooling.

**Figure 13:**
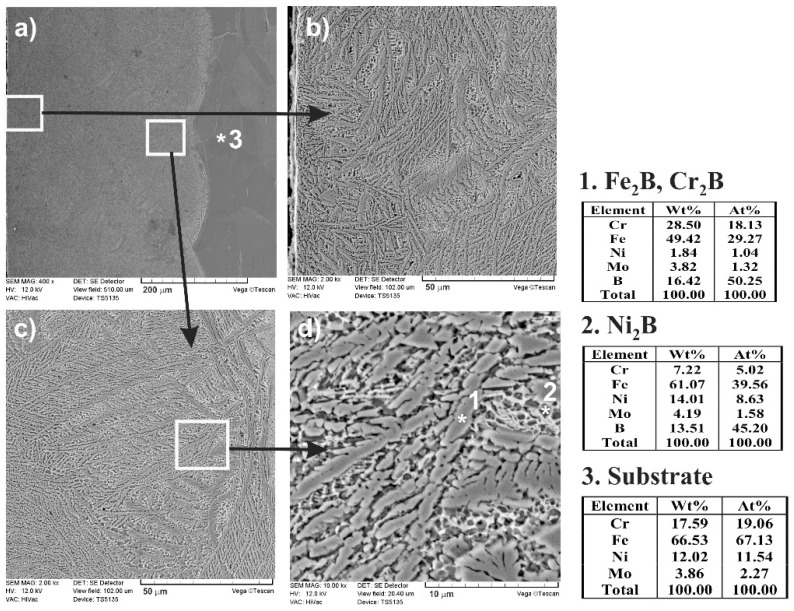
SEM microstructure of laser-alloyed layer with boron and Stellite-6, produced on austenitic 316L steel with a dilution ratio of 0.48 using laser beam power *P* = 1.56 kW; the entire re-melted zone (**a**), the area close to the surface with a high percentage of borides (**b**), the area close to the boundary between MZ and substrate material (**c**), Fe_2_B and Cr_2_B borides in the form of polygons or needle-shaped sticks as well as darker and finer Ni_2_B phase in the austenitic matrix at the boundary between MZ and substrate material (**d**).

The composite nature of the laser-alloyed layer with boron and Stellite-6 was clearly visible in SEM images, which were observed using a scanning electron microscope (Figure 13). The entire surface layer is presented in Figure 13a. The separate images taken at the higher magnification are shown in Figure 13b–d. The relatively high percentage of borides was characteristic of the region, occurring close to the surface (Figure 13b). However, at the boundary between the re-melted zone and the substrate material (Figure 13c,d), the percentage of the hard Fe_2_B and Cr_2_B borides, appearing in the form of polygons or needle-shaped sticks, as well as Ni_2_B phase (the darker and finer precipitates), was only slightly lower. The point X-ray microanalysis by EDS method indicated the presence of Fe_2_B or Cr_2_B borides as well as Ni_2_B borides in the austenitic matrix (the spots 1 and 2, respectively). The results from spot 3 were typical of the austenitic substrate material.

**Figure 14:**
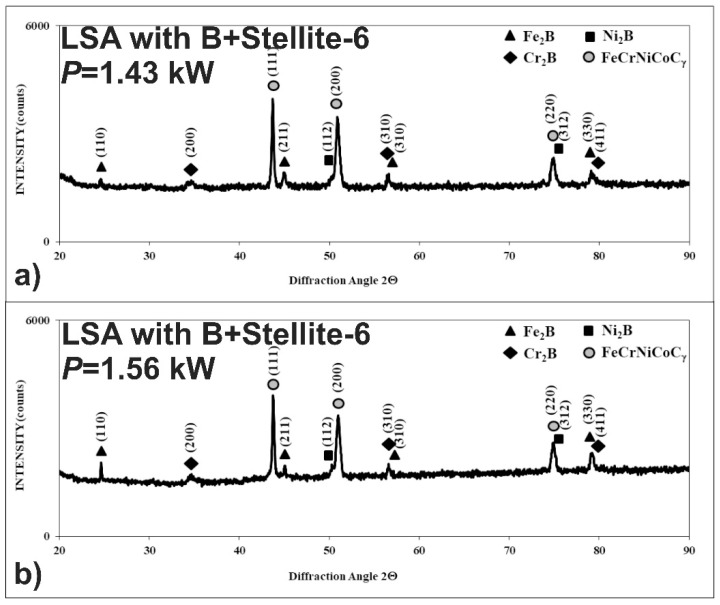
XRD patterns of the laser-alloyed layer with boron and Stellite-6, produced on austenitic 316L steel: with a dilution ratio of 0.41 using laser beam power *P* = 1.43 kW (**a**), with a dilution ratio of 0.48 using laser beam power *P* = 1.56 kW (**b**).

After LSA with boron and Stellite-6, the phase analysis was also performed by X-ray diffraction in order to identify the phases appearing in MZ. The XRD patterns are shown in Figure 14 in the case of using the laser beam power, both of 1.43 and 1.56 kW. They confirmed the presence of hard iron, chromium, and nickel borides (Fe_2_B, Cr_2_B, Ni_2_B) in a soft matrix, composed of alloyed austenite FeCrNiCoCγ. The possible peaks from the cobalt borides weren’t identified. Probably, cobalt occurred only in the alloyed austenite as an element dissolved in this phase. The significant differences in XRD patterns from the laser-alloyed layers, produced using various laser beam powers, weren’t observed.

**Figure 15:**
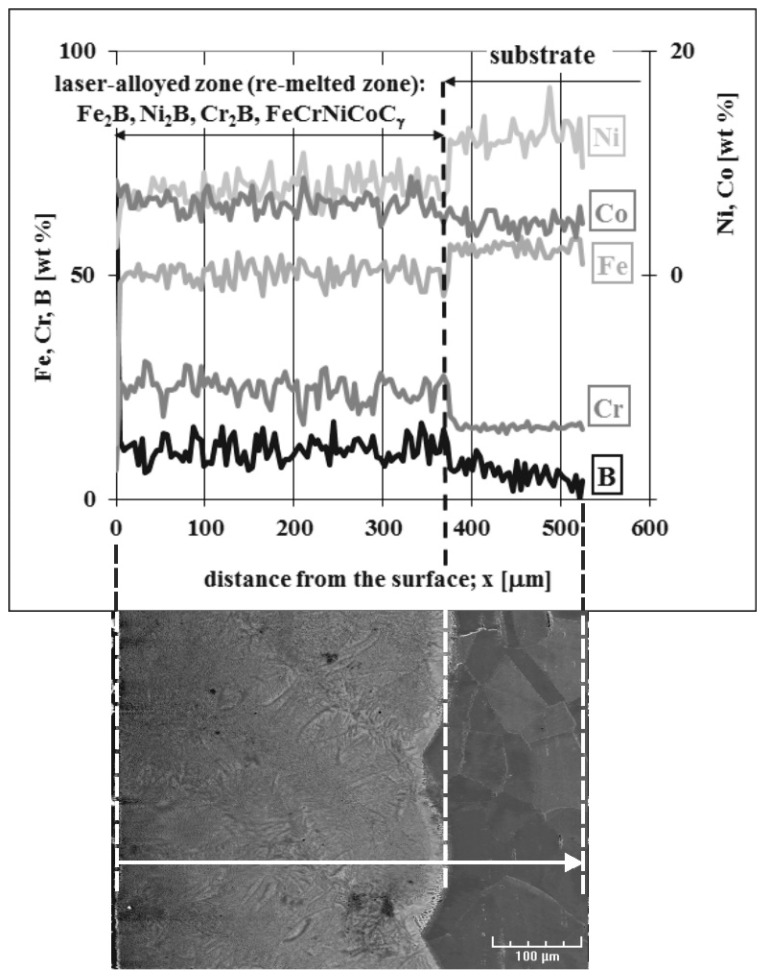
Results of linear X-ray microanalysis (EDS) of the laser-alloyed layer with boron and Stellite-6, produced on austenitic 316L steel with a dilution ratio of 0.48 using laser beam power *P* = 1.56 kW.

The concentration profiles of selected elements in the laser-alloyed layer with boron and Stellite-6, as well as in the substrate material, were studied using X-ray microanalysis by the EDS method. The SEM image of the microstructure, the line of measurements, and the linear profiles of elements are presented in Figure 15. LSA was carried out with a dilution ratio *DR* = 0.48 using a laser beam power of 1.56 kW. The main elements, occurring in austenitic 316L steel as well as in alloying material (i.e., Fe, Cr, Ni, Co, and B), were subjected to linear X-ray microanalysis. This time, the analysis of the molybdenum profile was omitted, taking into account the absence of molybdenum borides in the laser-alloyed layers with a dilution ratio above 0.37.

Like in the previous case (laser-borided layer), the diminished average values of iron and nickel concentrations were observed in the MZ compared to the substrate material. It resulted from the increased boron content in this region. Nickel and iron bonded to boron, creating Ni_2_B and Fe_2_B borides with their relatively high percentage in the re-melted zone. The increased content of boron was usually accompanied by diminished concentrations of iron and nickel. This indicated the presence of iron or nickel borides. On the contrary, the average concentration of chromium in the re-melted zone was higher in comparison with the substrate. It could result from the limited bonding of chromium and boron, confirming the relatively low percentage of chromium borides in the laser-alloyed layer. The low chromium contents and simultaneously high boron concentrations indicated the areas in which the chromium borides occurred. The significant chromium content remained in the austenitic matrix. Such a situation corresponded to the areas with high chromium concentrations. The diminished percentage of the austenitic matrix in the MZ was the reason for the increased chromium content in these regions. The profile of cobalt concentration indicated the increased mean cobalt content in the re-melted zone when compared to the substrate material. However, XRD didn’t confirm the presence of cobalt borides. Hence, all the cobalt content occurred in the austenitic matrix, i.e., FeCrNiCoCγ phase.

### 3.4. The Microstructure of Laser-Alloyed Layer with Boron and Nickel

**Figure 16:**
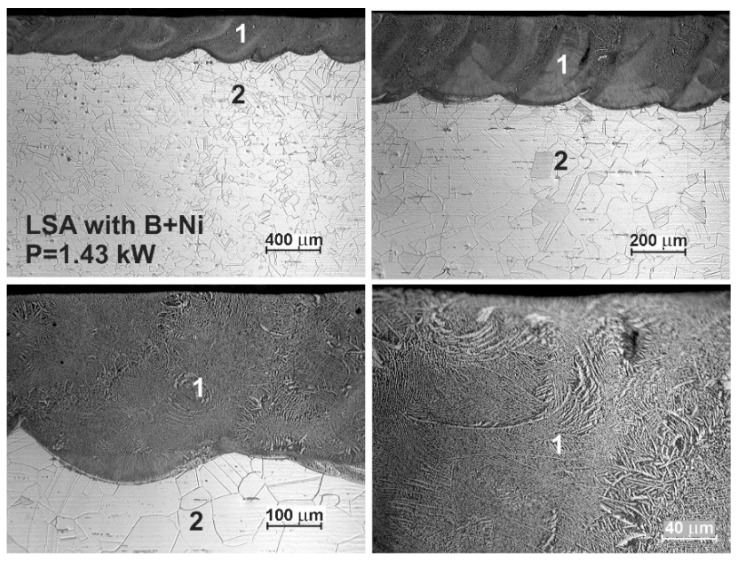
OM microstructure of laser-alloyed layer with boron and nickel, produced on austenitic 316L steel with a dilution ratio of 0.42 using laser beam power *P* = 1.43 kW; 1—re-melted zone (MZ), i.e., laser-alloyed layer, 2—substrate material (316L steel).

**Figure 17:**
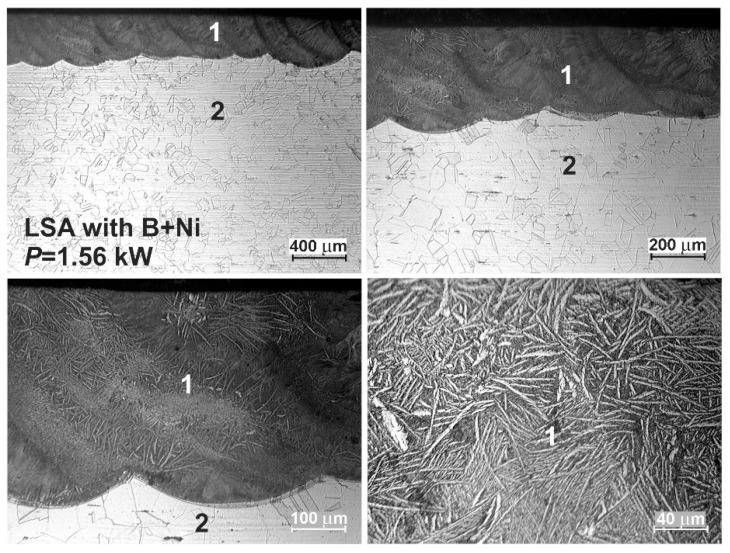
OM microstructure of laser-alloyed layer with boron and nickel, produced on austenitic 316L steel with a dilution ratio of 0.48 using laser beam power *P* = 1.56 kW; 1—re-melted zone (MZ), i.e., laser-alloyed layer, 2—substrate material (316L steel).

The OM microstructures of the laser-alloyed layers with a mixture of boron and nickel of mass ratio 1:1 are shown in Figure 16 and Figure 17. The re-melted zone, constituting a laser-alloyed layer, was formed using a laser beam power of 1.43 and 1.56 kW, respectively. The rest of the important laser processing parameters were constant, that is, laser beam diameter *d* = 2 mm, scanning rate *v*_l_ = 2.88 m·min^−1^, and overlapping *O* = 86%. The thickness of the preplaced coating with alloying material was also the same in both cases (200 µm). The use of the lower laser beam power (*P* = 1.43 kW) resulted in the formation of the surface layer with a dilution ratio *DR* = 0.42 and the average depth (*d*_MZ_) of 345 µm. The increase in the laser beam power up to 1.56 kW obtained the higher dilution ratio (*DR* = 0.48) as well as the average depth of MZ (*d*_MZ_ = 383 µm).

Similar to previous cases, the two zones were observed after the laser surface alloying: re-melted zone (1), i.e., laser-alloyed layer, and austenitic substrate (2). The MZ was characterized by the uniform depth throughout the whole cross-section due to the high overlapping used. The produced surface layer of very good quality was free of microcracks and gas pores (see Figure 16 and Figure 17). Like previously, there were no differences in the microstructure of the heat-affected zone and substrate material. It was, therefore, difficult to determine the extent of the HAZ.

**Figure 18:**
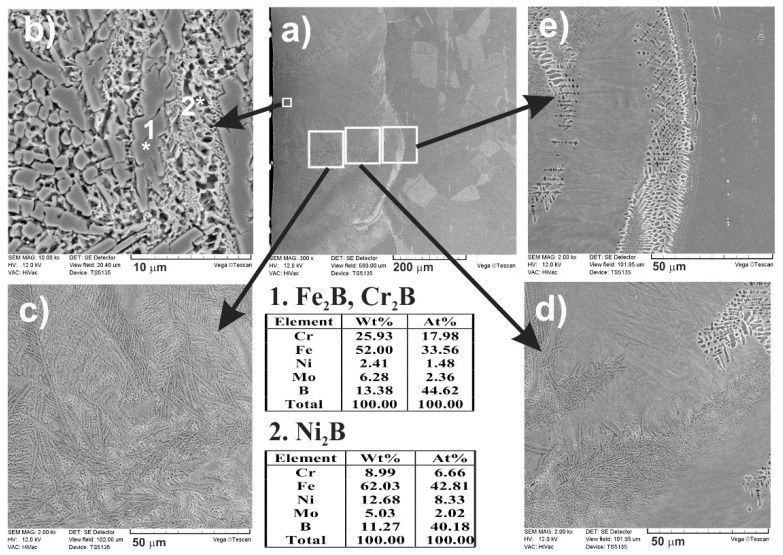
SEM microstructure of laser-alloyed layer with boron and nickel, produced on austenitic 316L steel with a dilution ratio of 0.42 using laser beam power *P* = 1.43 kW; the entire re-melted zone (**a**), the area close to the surface with Fe_2_B and Cr_2_B borides in the form of polygons or needle-shaped sticks as well as darker and finer Ni_2_B phase in the austenitic matrix (**b**), the area in the middle of MZ with, still, a high percentage of borides (**c**), the area close to the boundary between MZ and substrate material with a diminished percentage of borides (**d**), the boundary between MZ and substrate (**e**).

**Figure 19:**
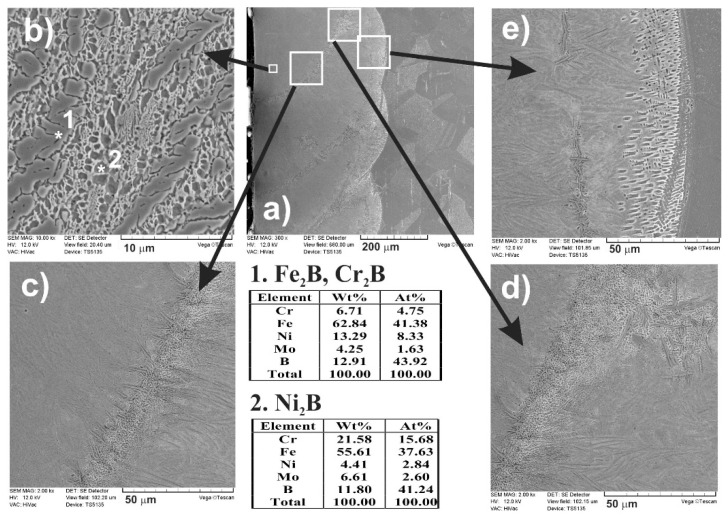
SEM microstructure of laser-alloyed layer with boron and nickel, produced on austenitic 316L steel with a dilution ratio of 0.48 using laser beam power *P* = 1.56 kW; the entire re-melted zone (**a**), the area close to the surface with Fe_2_B and Cr_2_B borides in the form of polygons or needle-shaped sticks as well as darker and finer Ni_2_B phase in the austenitic matrix (**b**), the area in the middle of MZ with, still, a high percentage of borides (**c**), the area close to the boundary between MZ and substrate material with a diminished percentage of borides (**d**), the boundary between MZ and substrate (**e**).

The SEM images (Figure 18 and Figure 19), taken with the scanning electron microscope, revealed the composite nature of the laser-alloyed layers, produced at the laser beam power of 1.43 and 1.56 kW, respectively. The hard borides were visible in the soft austenitic matrix. The diminished percentage of borides was observed with the increase in the distance from the laser-alloyed surface. Like in the previous cases, Fe_2_B and Cr_2_B borides appeared in the form of polygons or needle-shaped sticks, whereas the finer Ni_2_B phase occurred in the darker areas (Figure 18b and Figure 19b). It was confirmed by point X-ray microanalysis using the EDS method (see the results from spots 1 and 2).

**Figure 20:**
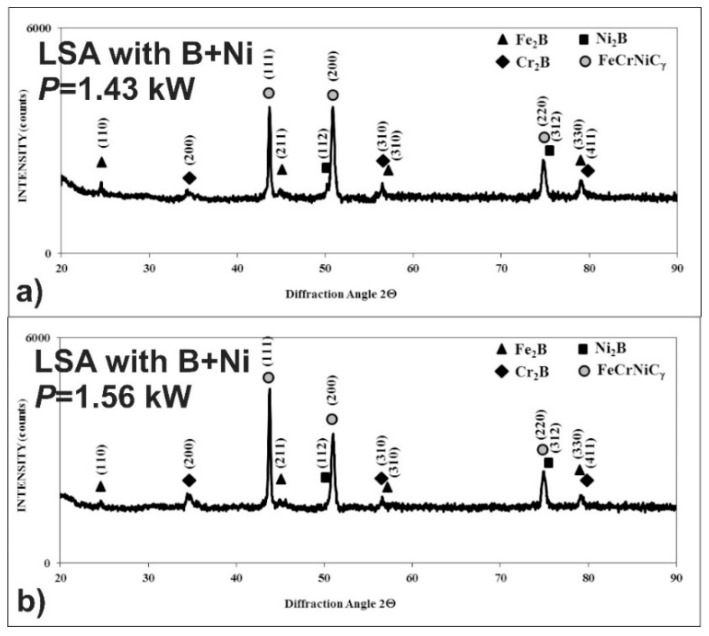
XRD patterns of the laser-alloyed layer with boron and nickel, produced on austenitic 316L steel: with a dilution ratio of 0.42 using laser beam power *P* = 1.43 kW (**a**), with a dilution ratio of 0.48 using laser beam power *P* = 1.56 kW (**b**).

After LSA of AISI 316L steel with boron and nickel, the phase analysis by XRD was also performed. In the case of laser processing using the laser beam power of both 1.43 and 1.56 kW, the peaks from an austenitic matrix (FeCrNiCγ phase) as well as hard iron, nickel, and chromium borides (Fe_2_B, Cr_2_B, Ni_2_B) were identified in the laser-alloyed layers (Figure 20). Probably, the use of the higher laser beam power of 1.56 kW resulted in the increased percentage of austenite and a diminished percentage of borides in such a composite surface layer because of the higher dilution ratio. This conclusion was confirmed by the intensity of peaks, corresponding to the considered phases.

**Figure 21:**
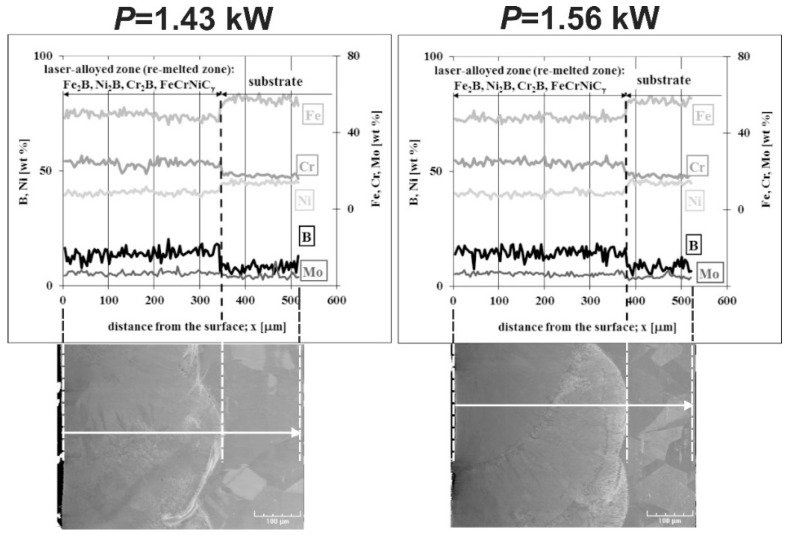
Results of linear X-ray microanalysis (EDS) of the laser-alloyed layer with boron and nickel, produced on austenitic 316L steel with a dilution ratio of 0.42 using laser beam power *P* = 1.43 kW (as well as with a dilution ratio of 0.48 using laser beam power *P* = 1.56 kW).

Linear X-ray microanalysis by EDS method was used in order to determine the concentration profiles of the selected elements in laser-alloyed layers with boron and nickel. The SEM images of laser-alloyed layers with marked lines of measurements and corresponding concentration profiles are shown in Figure 21. The analysis was performed, taking into account the most important elements of the austenitic 316L steel, as well as the elements constituting alloying material, i.e., Fe, Cr, Ni, Mo, and B. The measured concentration profiles of the considered elements after LSA using both *P* = 1.43 kW (left part of Figure 21) and *P* = 1.56 kW (right part of Figure 21) were similar to those obtained in the case of the laser-alloyed layer with only boron (Figure 10). In the re-melted zone, regardless of the dilution ratio used (0.42 or 0.48), the reduced contents of iron and nickel were measured in comparison with the substrate material. It could result from the increased concentration of boron and bonding of boron and iron or nickel to form corresponding borides of a relatively high percentage in MZ. The high boron concentrations were usually accompanied by relatively low contents of iron and nickel. It could indicate the presence of complex (Fe,Ni)_2_B borides. A fairly good correlation was also found between the iron and chromium concentration profiles. Therefore, the occurrence of complex phase (Fe,Cr)_2_B was also likely. However, the averaging chromium content in the re-melted zone was higher than that measured in the substrate. This was due to the reduced tendency of chromium to bond to boron and resulted in a relatively small percentage of chromium borides in the laser-alloyed layer. The low chromium concentrations, accompanied by high contents of boron, indicated the possible areas with the presence of chromium borides. However, much of the chromium remained in the austenitic matrix. The reduced percentage of alloyed austenite in the re-melted zone was the reason for the increased mean chromium concentration in this area. The mean concentration of molybdenum in MZ was also increased compared to the substrate. It could also indicate a limited ability of molybdenum to bond to boron. Hence, the molybdenum tended to remain in the austenitic matrix, not forming borides.

### 3.5. Microstructure of Laser-Alloyed Layer with Boron, Nickel and Chromium

**Figure 22:**
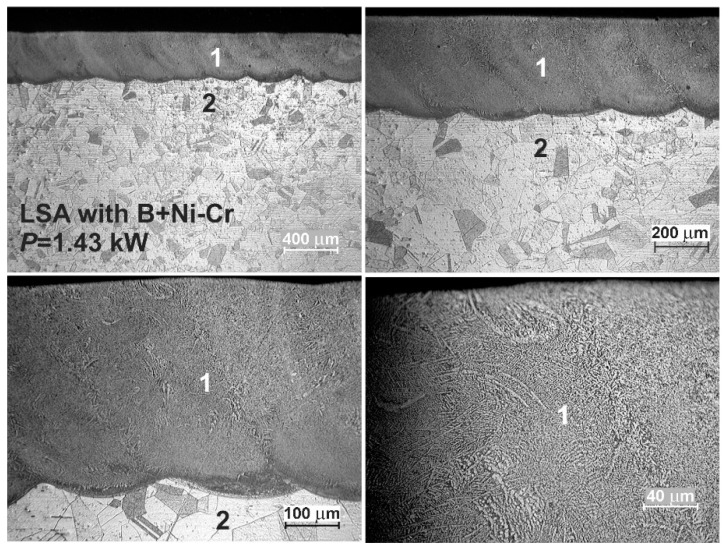
OM microstructure of laser-alloyed layer with boron, nickel, and chromium, produced on austenitic 316L steel with a dilution ratio of 0.43 using laser beam power *P* = 1.43 kW; 1—re-melted zone (MZ), i.e., laser-alloyed layer, 2—substrate material (316L steel).

**Figure 23:**
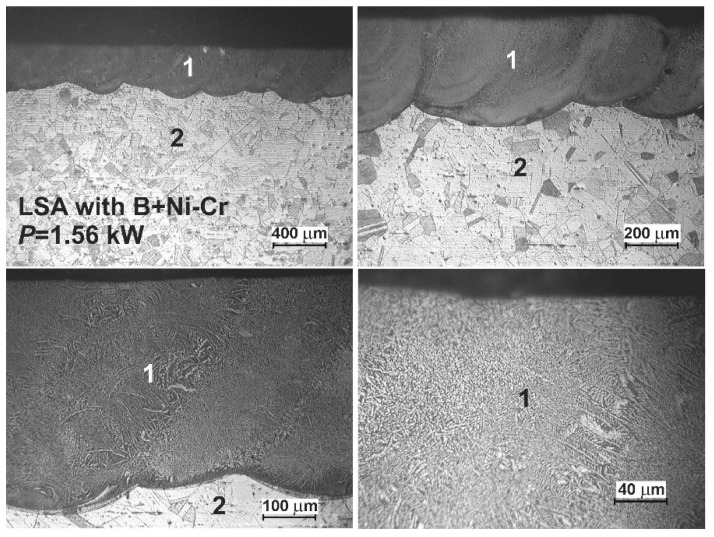
OM microstructure of laser-alloyed layer with boron, nickel, and chromium, produced on austenitic 316L steel with a dilution ratio of 0.49 using laser beam power *P* = 1.56 kW; 1—re-melted zone (MZ), i.e., laser-alloyed layer, 2—substrate material (316L steel).

The OM images of the laser-alloyed layers with boron, nickel, and chromium using the two laser beam powers, i.e., 1.43 and 1.56 kW, are shown in Figure 22 and Figure 23, respectively. Amorphous boron and a mixture of Ni and Cr powders (Ni–Cr) were used as an alloying material with a mass ratio of 1:1 (B:Ni–Cr) during LSA of 316L steel. Whereas the mass ratio of nickel to chromium (Ni:Cr) was equal to 4:1. The thickness of the preplaced coatings with alloying material (*t*_C_) was the same in both cases, obtaining about 200 μm. The averaging depth of the laser-alloyed layer (*d*_MZ_), produced at 1.43 kW, was equal to 352 μm, resulting in a dilution ratio (*DR*) of 0.43. Whereas the use of laser beam power of 1.56 kW caused an increase in the averaging depth of re-melted zone up to *d*_MZ_ = 395 μm and dilution ratio up to *DR* = 0.49. The rest of the parameters of the laser processing were the same as those used during LSA with other alloying materials, i.e., laser beam diameter *d* = 2 mm, scanning rate *v*_l_ = 2.88 m·min^−1^, and overlapping *O* = 86%.

As in previous cases, the two zones were observed in laser-alloyed 316L steel: a re-melted zone (1) with a fairly uniform thickness and an austenitic substrate (2). The MZ (1) was free of microcracks and gas pores (see Figure 22 and Figure 23). The differences in the microstructure of the heat-affected zone and the substrate material weren’t visible because of the reasons mentioned previously. Hence, the composite surface layers were composed of only laser-alloyed zones, i.e., laser re-melted zones, like in the previous LSA processes.

**Figure 24:**
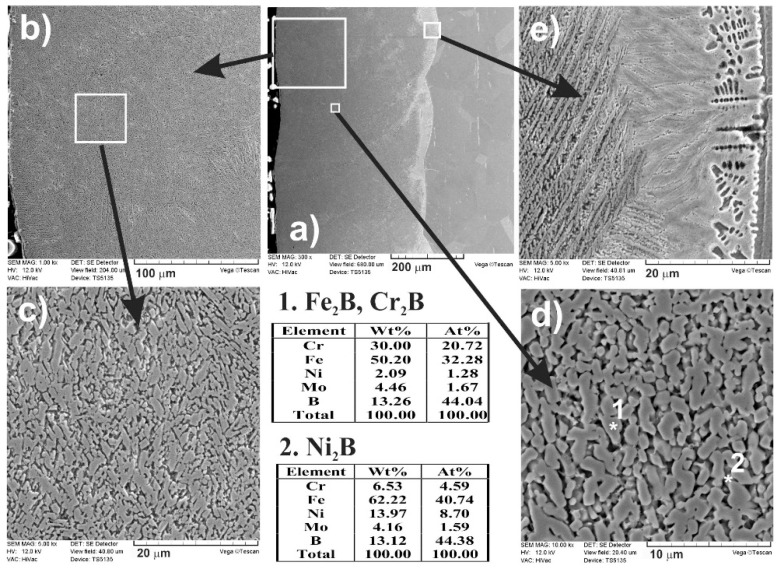
SEM microstructure of laser-alloyed layer with boron, nickel, and chromium, produced on austenitic 316L steel with a dilution ratio of 0.43 using laser beam power *P* = 1.43 kW; the entire re-melted zone (**a**), the area close to the surface with borides in the austenitic matrix (**b**) and (**c**), the area in the middle of MZ with, still, a high percentage of Fe_2_B and Cr_2_B borides in the form of polygons or needle-shaped sticks as well as darker and finer Ni_2_B phase (**d**), the area close to the boundary between MZ and substrate material with a diminished percentage of borides (**e**).

**Figure 25:**
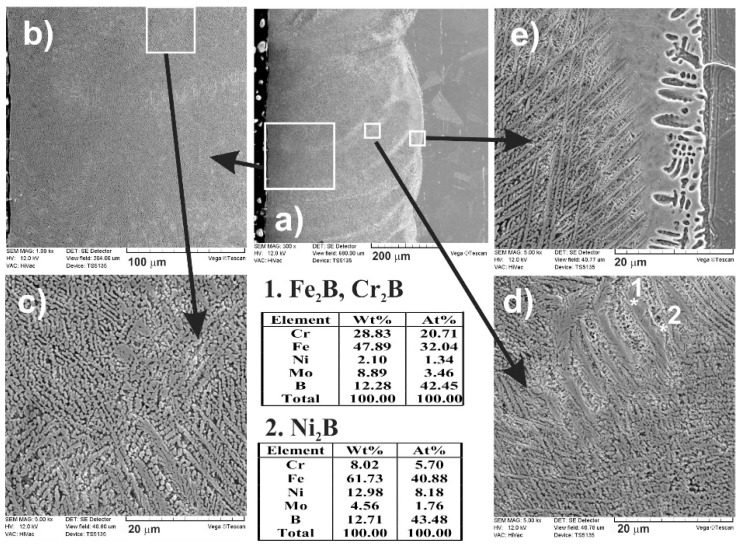
SEM microstructure of laser-alloyed layer with boron, nickel, and chromium, produced on austenitic 316L steel with a dilution ratio of 0.49 using laser beam power *P* = 1.56 kW; the entire re-melted zone (**a**), the area close to the surface with borides in the austenitic matrix (**b**) and (**c**), the area in the middle of MZ with, still, a high percentage of Fe_2_B and Cr_2_B borides in the form of polygons or needle-shaped sticks as well as darker and finer Ni_2_B phase (**d**), the area close to the boundary between MZ and substrate material with a diminished percentage of borides (**e**).

As previously, when boron, nickel, and chromium were used as an alloying material, SEM images (Figure 24 and Figure 25) obtained by scanning electron microscope also indicated the composite nature of laser-alloyed layers produced at the laser beam power of 1.43 and 1.56 kW, respectively.

The relatively high amount of hard metal borides occurred in the soft austenitic matrix close to the surface (Figure 24c and Figure 25c). It was clearly visible that the percentage of borides slightly diminished with the increasing distance from the surface (see Figure 24d,e and Figure 25d,e). Close to the boundary of the re-melted zone and the substrate material (Figure 24e and Figure 25e), the percentage of alloyed austenite in microstructure was the highest. Like after the previous LSA processes, Fe_2_B and Cr_2_B borides appeared in the form of polygons or needle-shaped sticks, and a finer Ni_2_B phase occurred in the darker areas (Figure 24d and Figure 25d). The elements’ concentrations, measured by point X-ray microanalysis using the EDS method, indicated the presence of iron and chromium borides as well as nickel borides in spots 1 and 2, respectively.

**Figure 26:**
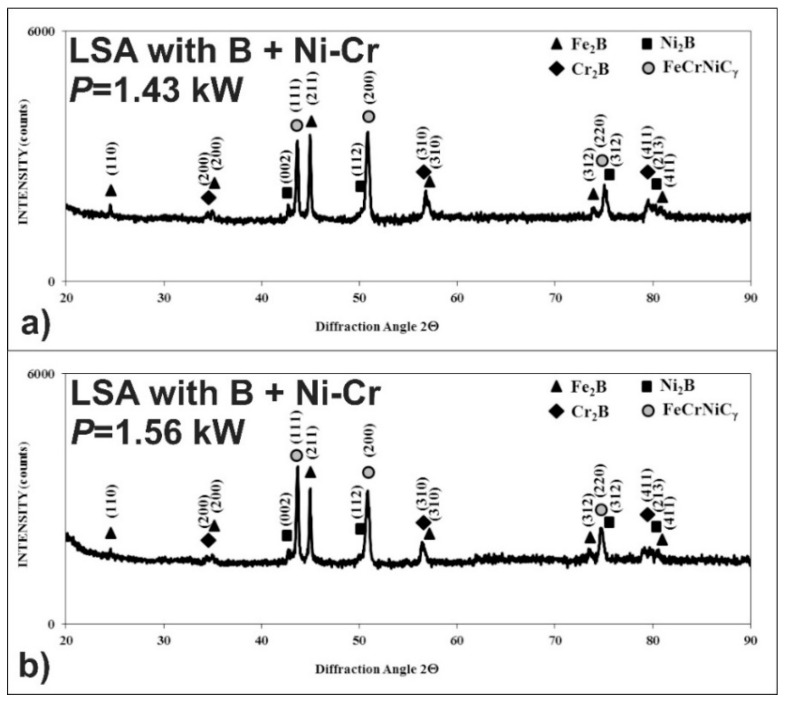
XRD patterns of the laser-alloyed layer with boron, nickel, and chromium, produced on austenitic 316L steel: with a dilution ratio of 0.43 using laser beam power *P* = 1.43 kW (**a**), with a dilution ratio of 0.49 using laser beam power *P* = 1.56 kW (**b**).

The phase analysis by XRD (Figure 26) confirmed the formation of the composite microstructure of MZ, consisting of hard ceramic phases (Fe_2_B, Cr_2_B, and Ni_2_B borides) in the austenitic matrix (FeCrNiCγ). Similar results were obtained in the case of using the laser beam power both of 1.43 kW (Figure 26a) and 1.56 kW (Figure 26b). XRD patterns also showed the effect of the laser beam power and dilution ratio on the intensity of peaks derived from alloyed austenite and metal borides. The higher laser beam power and, as a consequence, higher dilution ratio (Figure 26b) resulted in the increased intensity of the peaks from austenite and the diminished intensity of peaks from metal borides. Based on these observations, it was probable that the percentage of hard borides in the laser-alloyed layer was diminished in the case of using the laser beam power of 1.56 kW.

**Figure 27:**
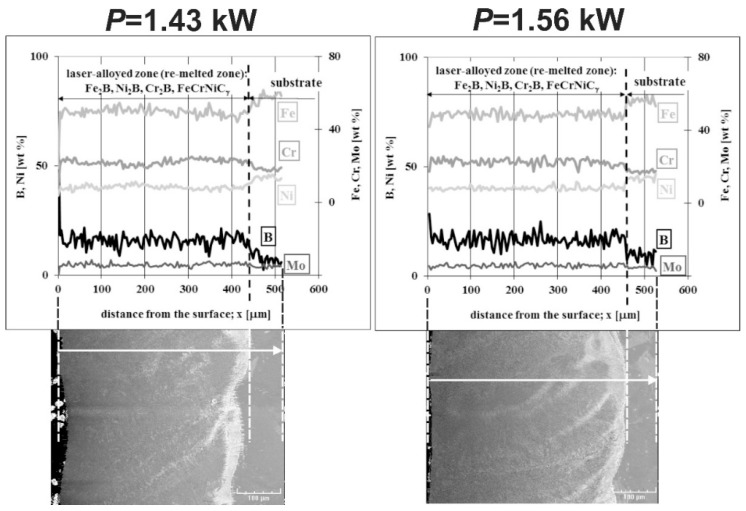
Results of linear X-ray microanalysis (EDS) of the laser-alloyed layer with boron, nickel, and chromium, produced on austenitic 316L steel with a dilution ratio of 0.43 using laser beam power *P* = 1.43 kW, as well as with a dilution ratio of 0.49 using laser beam power *P* = 1.56 kW.

The concentration profiles of selected elements in laser-alloyed layers were studied using linear X-ray microanalysis by the EDS method. The SEM images of microstructures together with the lines of measurements and the obtained distribution of elements across the composite surface layers are presented in Figure 27. The most important elements of the 316L steel and alloying material (i.e., Fe, Cr, Ni, Mo, and B) were analyzed. The two laser beam powers were taken into account: 1.43 kW (left part of Figure 27) and 1.56 kW (right part of Figure 27). In general, the determined concentration profiles didn’t differ from those measured in the case of laser-alloyed 316L steel exclusively with boron (Figure 10) or with boron and nickel (Figure 21). After LSA, at the laser beam power of both 1.43 and 1.56 kW, the diminished averaging concentrations of iron and nickel were observed in MZ compared to the substrate material. Like previously, it resulted from the creation of iron and nickel borides (Fe_2_B and Ni_2_B) in this area because of the increased boron content. The high boron concentration was usually accompanied by decreased contents of iron and nickel. A good correlation of iron and nickel profiles could indicate the possible formation of the complex (Fe,Ni)_2_B borides. Simultaneously, the chromium concentration profile was fairly compatible with the iron profile. This revealed the possible presence of complex (Fe,Cr)_2_B borides. However, the averaging chromium content in the laser-alloyed layer (i.e., MZ) was increased compared to the concentration of this element in the substrate. Probably, it resulted from the limited ability to form chromium borides, as was explained above. The relatively low chromium content usually corresponded to the high boron concentration. In such areas, chromium borides could occur. However, much of the chromium remained in the austenitic matrix, and the reduced percentage of alloyed austenite in the re-melted zone resulted in an increase in the average chromium concentration in that area. The molybdenum concentration profile also indicated an increased average content of this element in the MZ compared to the substrate. This could provide a conclusion that there was a limited ability to form molybdenum borides. Accordingly, molybdenum tended to remain in an austenitic matrix.

### 3.6. Cohesion of Laser-Alloyed Layers

The evaluation of the cohesion of the produced 316L steel laser-alloyed layers with only boron as well as boron and selected metallic elements was carried out by the Rockwell C hardness test, a test commonly used in studies of coatings’ adhesion. The indents were made on the surface of the laser-alloyed layers, and the defects around them were observed using an optical microscope.

**Figure 28:**
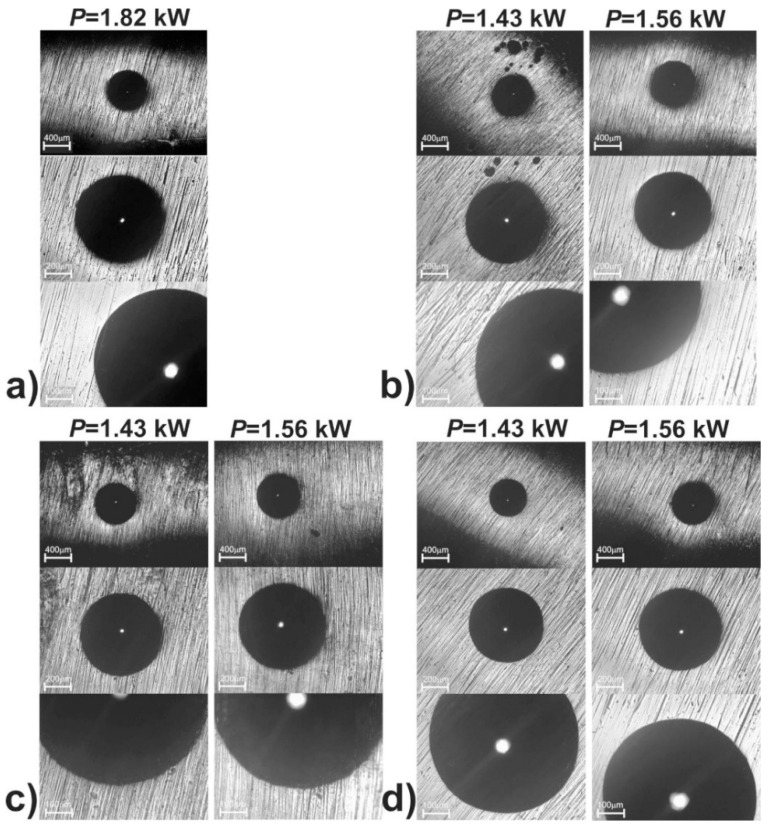
OM images of the indents, performed using the Rockwell C hardness test on the surface of laser-borided layer (**a**), laser-alloyed layer with boron and Stellite-6 (**b**), laser-alloyed layer with boron and nickel (**c**), laser-alloyed layer with boron, nickel, and chromium (**d**).

OM images of the surface of all the laser-alloyed layers with the visible indents are shown in Figure 28. No radial or circumferential cracks, as well as no delamination or spalling, were visible at the edges of the indents, performed on the surface of both the laser-borided layer (Figure 28a) and laser-alloyed layers with boron and selected metallic elements (Figure 28b–d). It was possible to assign the HF1 cohesion pattern for all the surface layers produced. The reason for the very good cohesion was undoubtedly the good quality of the microstructure of all the laser-alloyed layers (no cracks and gas pores) with a considerably greater dilution ratio (*DR*) than the required minimum value (0.37).

## 4. Conclusions

The adequate selection of parameters of the LSA with boron and some metallic elements resulted in the formation of surface layers on the austenitic 316L steel. The effects of such a treatment on the microstructure allowed to formulate the following conclusions:

- Surface layers produced by laser alloying of 316L steel with boron or boron and selected metallic elements (Ni, Cr, Co) were characterized by a microstructure of acceptable quality, i.e., devoid of defects typical of laser processing (microcracks, gas pores),
- Obtaining a layer without defects required a dilution ratio (*DR*) of at least 0.37. All the laser-alloyed layers were characterized by the relatively high dilution ratio in the range of 0.41–0.54,
- All the laser-alloyed layers constituted the re-melted zone (MZ) only,
- The effects of laser irradiation weren’t observed in the heat-affected zone (HAZ) due to no possibility of changing the austenitic structure by martensite transformation during fast cooling. Hence, the microstructure of HAZ didn’t differ from the substrate material,
- All the re-melted zones produced were of a composite nature, i.e., the hard ceramic phases, such as Fe_2_B, Cr_2_B, Ni_2_B, or Ni_3_B borides, occurred in the austenitic matrix,
- The use of powder mixtures of boron and selected metallic elements as alloying materials, instead of only boron powder, made it possible to reduce the laser beam power needed to produce the laser-alloyed layers of acceptable quality,
- The uniform thickness of each laser-alloyed layer, i.e., depth of re-melted zone, was achieved because of the relatively high overlapping (86%),
- The thickness of the laser-alloyed layers ranged from 308 to 432 μm and was much greater than the thickness of the surface layers produced on austenitic 316L steel by diffusion techniques of boriding,
- All the laser-alloyed layers, produced in austenitic 316L steel, were characterized by excellent cohesion,
- The LSA process with boron and the selected metallic elements was energy- and material-saving as well as environmentally friendly compared to the diffusion processes of boriding,
- The usefulness of the proposed laser surface alloying to increase hardness and wear resistance of 316L steel without sacrificing its corrosion resistance would be confirmed in the next paper.

## Figures and Tables

**Table 1:** The chemical composition of 316L steel.

Element	C	Cr	Ni	Mo	Mn	Si	Fe
(wt%)	0.023	17.45	12.92	2.88	0.56	0.45	balance

**Table 2:** The chemical composition of Stellite-6 powder.

Element	C	Cr	Ni	W	Mn	Si	Fe	Co
(wt%)	1.2	28.0	<3.0	4.5	0.56	1.1	<3.0	balance

**Table 3:** Parameters of laser surface alloying of 316L steel.

Type of Alloying Material	Thickness of Paste Coating *t*_C_ (mm)	Laser Beam Diameter *d* (mm)	Scanning Rate *v*_l_ (m·min^−1^)	Overlapping *O* (%)	Laser Beam Power *P* (kW)	Averaging Irradiance *E* (kW/cm^2^)
B	200	2	2.88	86	1.82	59.73
B + Stellite-6, mass ratio 1:1					1.43	45.52
					1.56	49.66
B + Ni, mass ratio 1:1					1.43	45.52
					1.56	49.66
B + Ni–Cr (4:1), mass ratio 1:1					1.43	45.52
					1.56	49.66

**Table 4:** The dilution ratio of laser-alloyed 316L steel.

Type of Alloying Material	Laser Beam Power *P* (kW)	Averaging Depth of MZ *d*_MZ_ (μm)	Dilution Ratio *DR*
B	1.82	432	0.54
B + Stellite-6, mass ratio 1:1	1.43	338	0.41
	1.56	384	0.48
B + Ni, mass ratio 1:1	1.43	345	0.42
	1.56	383	0.48
B + Ni–Cr (4:1), mass ratio 1:1	1.43	352	0.43
	1.56	395	0.49
